# National scale down‐the‐drain environmental risk assessment of oxybenzone in the United States

**DOI:** 10.1002/ieam.4430

**Published:** 2021-05-20

**Authors:** Emily E. Burns, Susan A. Csiszar, Kyle S. Roush, Iain A. Davies

**Affiliations:** ^1^ Personal Care Products Council Washington District of Columbia USA; ^2^ Procter & Gamble Cincinnati Ohio USA

**Keywords:** BP‐3, Down‐the‐drain freshwater aquatic risk assessment, Exposure modeling, UV filters

## Abstract

Organic ultraviolet (UV) filters are used in cosmetic and personal care products (CPCPs) and over‐the‐counter (OTC) sunscreens, due to their ability to absorb solar radiation. When OTC and CPCP ingredients are washed down the drain, they can then enter freshwaters that receive wastewater treatment plant effluents. This paper presents a freshwater environmental safety assessment of a key UV filter, oxybenzone, used in OTC sunscreens and CPCPs in the United States. Exposure was characterized using iSTREEM^®^, a spatially resolved aquatic exposure model developed for chemicals disposed of down the drain. iSTREEM^®^ provides a comprehensive exposure assessment of oxybenzone concentrations in United States receiving waters through predicted environmental concentration (PEC) distributions representative of conditions across the region. A review of available hazard data was used to derive a predicted no‐effect concentration (PNEC) using aquatic toxicity data and assessment factors. A safety assessment was conducted by comparing the PEC distribution with the PNEC. The results indicate that oxybenzone is of low concern and there is a significant margin of safety as the 90th percentile PEC is two orders of magnitude below the PNEC. These results are instrumental in demonstrating the environmental safety of key organic UV filters in the U.S. freshwater environment and will help prioritize future work. *Integr Environ Assess Manag* 2021;17:951–960. © 2021 Personal Care Products Council. *Integrated Environmental Assessment and Management* published by Wiley Periodicals LLC on behalf of Society of Environmental Toxicology & Chemistry (SETAC)

## KEY POINTS

- A freshwater environmental risk assessment for oxybenzone was conducted that considered available aquatic toxicity data and down‐the‐drain emissions of sunscreen, cosmetic, and personal care products.
- A nationwide distribution of predicted environmental concentrations was modeled, using the spatially explicit iSTREEM® exposure model, and the median and 90th percentile oxybenzone concentrations were predicted to be 0.01 and 0.15 µg/L, respectively.
- The 90th percentile exposure prediction (reasonable worst case) is two orders of magnitude below the predicted no‐effect concentration value of 18 µg/L, indicating that risk is negligible.
- This paper provides a down‐the‐drain freshwater environmental risk assessment framework suitable for over‐the‐counter drugs, cosmetic, and personal care ingredients.

## INTRODUCTION

There has been increasing public and research interest in the presence of ultraviolet (UV) filters in aquatic environments and the magnitude of risk they pose to exposed organisms. UV filters play an important role in sun safety regimes (i.e., application of over‐the‐counter [OTC] sunscreen products), as they absorb UV solar radiation, a risk factor for keratinocyte cancers and melanoma (Neale et al., 2021). Due to their UV‐protective properties, UV filters are also used as cosmetic and personal care product (CPCP) ingredients (Manová et al., 2013). UV filter exposure in the marine environment is driven by the wash‐off of sunscreens during recreational activities (e.g., bathing and swimming) (Labille et al., 2020; Mitchelmore et al., 2019). This has spurred several investigations into the exposure of coral reefs to UV filters and their ecotoxic potential; we direct the interested reader to the review by Mitchelmore et al. (2021). In response, local and state lawmakers in Palau (Remengesau, 2018), the U.S. Virgin Islands (U.S. Virgin Islands, 2019), and most recently, Hawaii (State of Hawaii Senate, 2018) banned the sale and distribution of specific UV filters in OTC products, in particular oxybenzone (BP‐3). This ban was enacted before the completion of a robust environmental risk assessment (ERA) and risk–benefit analyses. Recently, the U.S. National Academies of Sciences, Engineering, Medicine was tasked by the U.S. Environmental Protection Agency (USEPA) with determining data gaps and/or risks UV filters pose to both the freshwater and marine environment and to assess the impact on public health due to potential changes in sunscreen use (NASEM, 2020).

Although the focus on the environmental impacts of UV filters has been on the marine environment (due to sunscreens use at beaches), UV filters are also used daily in CPCPs for which the main environmental exposure route is wastewater treatment plant (WWTP) effluents discharged to freshwaters. A review of UV filter concentrations in rivers and WWTP effluent across the globe reveals that BP‐3 is indeed released to and is present in this matrix (e.g., Česen et al., 2019; Kameda et al., 2011; Kasprzyk‐Hordern et al., 2009; see Table S6). Current global monitoring data suggest that concentrations in freshwater are generally below 0.1 µg/L (e.g., across 42 data points globally, Table S6). Freshwater ERAs focusing on BP‐3 have been published in other regions using this type of literature‐based environmental exposure dataset (e.g., Carve et al., 2021; Guo et al., 2020; Kim & Choi, 2014). However, there is no ERA for BP‐3 in the United States. Therefore, the aim of this study is to conduct a down‐the‐drain U.S. freshwater ERA on a UV filter of recent regulatory concern, BP‐3, used in OTC sunscreens and CPCPs.

Environmental risk assessment is a fundamental component of environmental chemical management. Standardized approaches are available for effluent‐receiving freshwaters where exposure is characterized as a predicted environmental concentration (PEC) and compared with a predicted no‐effect concentration (PNEC). Standardized ERA follows a tiered approach such that lower tiers employ conservative assumptions and, if negligible risk is identified, strongly suggests the chemical is unlikely to pose an unacceptable risk to the environment (Nabholz, 1991) and further work is not prioritized. If an unacceptable risk is identified in a lower tier, higher tier refinements can be made, mainly through the collection of additional data, before concluding on risk (Salvito et al., 2002; USEPA, 1998).

The review of global freshwater monitoring data yielded few U.S. measurements and these data are specific to recreational direct discharge emissions rather than down‐the‐drain emissions (see Table S6); therefore, limited U.S.‐specific demographic, infrastructure, emission, and environmental trends have been included. To fill this gap, exposure modeling was used for the exposure characterization. Exposure modeling is a rapid, yet conservative, and reliable method that is ideal for a U.S. national scale exposure assessment of down‐the‐drain CPCP and OTC ingredients. When monitoring data are not available, the use of exposure models to derive PECs is standard in proactive and protective ERAs, including regulatory ERA frameworks in the United States and European Union (EC JRC, 2003; Zeeman & Gilford, 1993). One model that is used to estimate U.S.‐wide PECs is the iSTREEM® model (Kapo et al., 2016), a well‐established, spatially explicit aquatic exposure model specifically designed to evaluate the fate and corresponding aquatic exposure from down‐the‐drain emissions of U.S. consumer chemicals and has been used for ERA (Aronson et al., 2012; Federle et al., 2014; McDonough et al., 2016; McDonough, Casteel, et al., 2017; Simonich et al., 2013). It has been demonstrated to predict conservative, yet realistic, surface water concentrations across the conterminous United States for consumer chemicals from down‐the‐drain sources (Kapo et al., 2016). Therefore, it is an appropriate model for predicting U.S. surface water concentrations of down‐the‐drain BP‐3 for ERA.

A suite of measured effects data is available for BP‐3 and covers both acute and chronic endpoints across several freshwater aquatic species. The species covered represent the three key taxa used in standard freshwater ERA approaches (EC JRC, 2003; Nabholz, 1991) and comprise a full acute and chronic dataset across these trophic levels (fish, invertebrate, algal). These data were evaluated and the most sensitive chronic toxicity response was selected as the endpoint from which to derive a robust PNEC with an appropriate assessment factor based on available toxicity data.

This paper presents a detailed review of BP‐3 hazard data and a robust exposure modeling assessment, which were synthesized into a U.S.‐specific risk characterization by comparing the modeled PEC distribution with the derived PNEC. These results are then compared with freshwater ERAs for BP‐3 from other regions. This paper also provides a framework for down‐the‐drain risk assessment of OTC and CPCP ingredients in the United States, which can also be applied globally.

## METHODS

The scope of the study is to assess the environmental safety of BP‐3 released down‐the‐drain from OTC sunscreens and CPCPs in the United States. As the emissions will predominantly flow to WWTPs and be released to their receiving waters, safety will be assessed in this compartment. The ERA consists of a review of the chemical properties, followed by an exposure assessment, hazard assessment, and risk characterization. A detailed overview of the ERA methodology is provided in Figure S1.

### Chemical properties

On the basis of the modeling approach applied in this study, the key physicochemical properties required for BP‐3 are solubility (6 mg/L, measured) and the log *K*_ow_ (3.45, measured) (ECHA, 2020). A biodegradation screening test showed that BP‐3 is biodegradable, as it reached 62% biodegradation in 28 days after an 11‐day lag period (not meeting the 10‐day window) in a manometric respirometry ready biodegradation test (retrieved from ECHA 2020). Further physicochemical properties are summarized in Table S1.

### Exposure characterization

**Figure 1:**
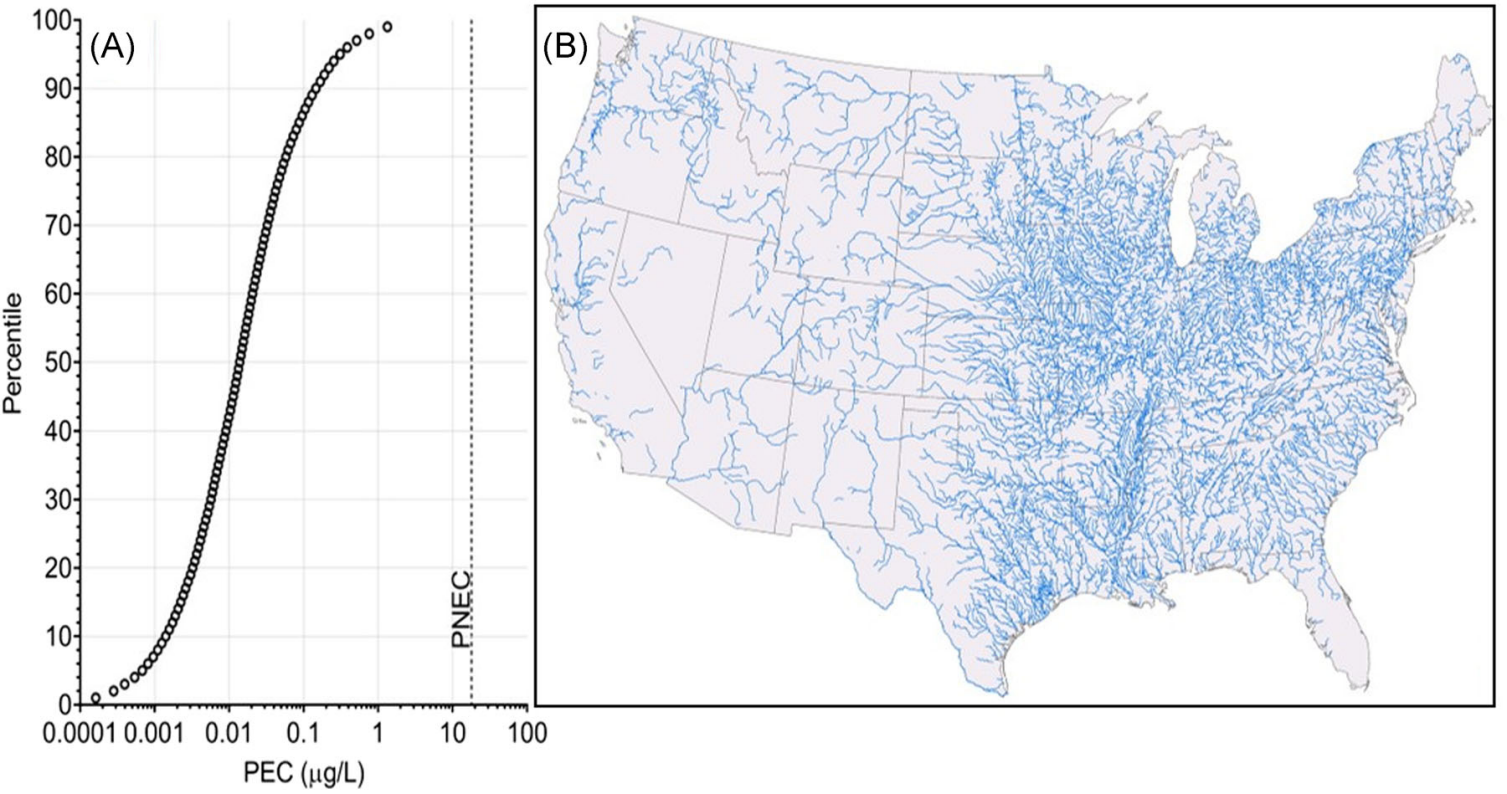
(A) Distribution of modeled oxybenzone concentrations per river segment (*n* = 227 876). The PNEC (18 µg/L) is plotted as a vertical dashed line for comparison. (B) Map of the river segments modeled across the United States; it should be noted that this map indicates the spatial extent of the rivers modeled and does not represent modeled concentrations. PEC, predicted environmental concentration; PNEC, predicted no‐effect concentration

Exposure was characterized using BP‐3 emissions and the iSTREEM® model to predict concentrations in river catchments across the United States. The model is a digitized U.S. river network comprised of 227 876 unique river segments (Figure 1B) for which river and chemical flows are connected. It uses the spatial location, size, and treatment type of 13 244 WWTPs as the source of chemical inputs to river segments, and the chemical concentration is estimated for each segment. Fate processes covered in iSTREEM® include WWTP removal and in‐stream decay (the latter due to biodegradation), both of which are chemical‐specific and input to the model. The model allows for national‐scale surface water concentrations to be represented as a concentration distribution considering spatial variability in chemical inputs and river flows.

The annual OTC sunscreen and CPCP emission of BP‐3 was estimated using 2019 market sales data from Euromonitor International, which have been used in previous ERA exposure assessments (Euromonitor, 2020; Hodges et al., 2014). Wash‐off was conservatively assumed to be 100% as estimated wash‐off fractions are typically not included in conservative ERAs, even though there are methods to estimate these fractions (Csiszar et al., 2016; Jolliet et al., 2015), and these approaches could be leveraged in the future if exposure refinement is needed. Annual BP‐3 tonnage was converted to grams emitted per capita per day (g/c/day) using a July 2019 US population of 328 239 523 (US Census Bureau, 2019), resulting in a final BP‐3 emission of 0.011 g/c/day. Although emission estimates are available on a yearly basis, there may be some seasonal variability in UV filter down‐the‐drain emissions, for example, higher usage in summer months. To explore the effects of this on modeled concentrations, the daily emissions were also doubled to represent a scenario where all BP‐3 would be emitted down the drain over a half‐year period. Although this is a conservative assumption as several daily‐use products contain UV filters, it provides insight into how yearly emission estimates can be interpreted within the risk assessment.

WWTP removal was characterized by averaging the activated sludge (AS) removal rates reported in the literature resulting in an AS removal of 86% (*n* = 21, Table S2). This WWTP removal is expected due to BP‐3 being biodegradable, as discussed above. The mean of the measured WWTP removal was leveraged for all iSTREEM® biological treatment input types: Oxidation ditch, lagoon, trickling filter, and rotating biological contractor, whereas primary removal was set to zero, similar to previous iSTREEM® modeling exercises (Aronson et al., 2012). As BP‐3 is biodegradable, it will also undergo in‐stream decay due to biodegradation. In‐stream decay was characterized by a generic first‐order rate constant (*k*) assigned according to the result of the biodegradation screening test (i.e., biodegradable, not meeting the 10‐day window), in this case, 0.014 day^−1^ (50‐day half‐life) (EC JRC, 2003). A summary of iSTREEM® inputs can be found in Table S3.

### Hazard characterization

The hazard assessment follows a standardized approach based on exposure of freshwater model species, evaluation of ecotoxicological endpoints, and the assignment of an appropriate assessment factor (AF) (ECHA, 2008; Nabholz, 1991). Hazard data for BP‐3 were collected from the European Chemicals Agency (ECHA) database (retrieved from ECHA, 2020) and published literature (summarized in Table S4). All studies were reviewed for reliability and relevance. Data related to ecologically relevant endpoints for risk assessment (e.g., mortality, growth, reproduction) from the studies conducted according to or similar to standardized test guidelines were selected for use in the hazard characterization. Although the median lethal and effect concentration (LC50, EC50) is commonly accepted as the acute aquatic toxicity endpoint of interest, there are several alternative toxicity endpoints that have been utilized in chronic aquatic exposures including the maximum acceptable toxicant concentration (MATC), no observed effect concentration (NOEC), lowest observed effect concentration, and effect concentration *x*% (EC*x*). Two of the most common and standard endpoints for use in ERA are the EC10 and the NOEC (ECHA, 2008; USEPA, 2004), and thus were used in the hazard assessment. The EC10 was the preferred endpoint, given the similar sensitivity and flaws associated with the NOEC (Beasley et al., 2015; Crane & Newman, 2000; ECHA, 2008). As such, EC10 was prioritized over the NOEC when available. The freshwater PNEC used for the risk characterization was calculated by application of an appropriate AF to the most sensitive endpoint (i.e., division of the lowest chronic value by the AF), following USEPA guidance (Beasley et al., 2018; Nabholz, 1991; Zeeman & Gilford, 1993; Table S5).

### Risk characterization

The safety assessment was conducted by comparing PEC percentiles (25th, 50th, 75th, and 90th) with the PNEC. The 50th percentile is the midpoint of the entire dataset (*n* = 227 846) where the data are most robust and not skewed by outliers produced by the model (Kapo et al., 2016). The 90th percentile was included as a “reasonable worst‐case” PEC, as it represents a concentration well into the tail of the distribution, particularly as the data follow a lognormal distribution (Kapo et al., 2016; McDonough et al., 2016). Comparing the 90th percentile with the PNEC is also consistent with regulatory ERA guidance, for example, the “worst‐case” exposure assessment outlined by Nabholz (1991) or the percentile used when distributional concentration data are available (EC JRC, 2003). Environmental risk is considered low, or negligible if the PEC is less than the PNEC (ECHA, 2012; Nabholz, 1991).

## RESULTS AND DISCUSSION

### PEC of oxybenzone

Predicted iSTREEM® mean‐flow concentrations in U.S. rivers from down‐the‐drain emissions of BP‐3 are presented as a distribution in Figure 1A. The 25th, 50th, 75th, and 90th percentile concentrations are summarized in Table 2 and they ranged from 0.004 to 0.15 µg/L. The percentile concentrations represent the spatial distribution of PECs. For example, the 50th percentile PEC is 0.01 µg/L, indicating that for 50% of the river segments, concentrations are below this level. To visualize the national extent of the data used to derive the concentration distributions, the segments comprising the river network underpinning the model are shown in Figure 1B. Each segment is influenced by a combination of spatially explicit parameters including river flow, BP‐3 loads transported from upstream, and WWTP discharges that correspond to both the size (e.g., population served) and treatment type of the 13 245 WWTPs included in the model (Figure S2).

The PEC can be regarded as conservative, as it is assumed that 100% of BP‐3 used annually in CPCPs and OTC sunscreens is released to wastewater. First, the 2019 emission estimate was compared against another reliable source for chemical volumes, IHS Markit (Mueller et al., 2020); the data were highly consistent with each other, indicating that emission estimates used are reasonable and appropriate. Second, we assumed that all BP‐3, which can also be used in sunscreens and washed‐off at beaches or lakes, will be washed entirely down the drain. UV filter measurements in waters near beaches and in the marine environment confirm that indeed some volume of sunscreens is directly discharged to nearshore waters rather than washed down the drain (Labille et al., 2020; Tashiro & Kameda, 2013). Third, Straub (2002) estimated that 10% of sun care products go to household waste, through the disposal of overaged products and residual amounts left in packaging. In a monitoring study in Korea, seasonal WWTP loading of BP‐3 was not observed as compared with other UV filters studied (Ekpeghere et al., 2016). As the iSTREEM® model is not seasonally specific, but rather representative of average yearly conditions (e.g., WWTP removal, in‐stream decay and flow), and available experimental evidence suggests seasonal loading of BP‐3 in WWTP influent is not pronounced, loading of BP‐3 emissions into half the year would likely result in overly conservative exposure predictions using our modeling approach. Therefore, the assumption that 100% of BP‐3 emissions go down the drain was determined to be suitably protective of temporal variations in usage and this is discussed further when comparing modeled with measured concentrations.

Environmental fate information was included to enhance environmental realism in exposure predictions (Salvito et al., 2002). For example, biodegradability screening tests are themselves more conservative (i.e., have stricter pass/fail criteria) as compared with higher tier biodegradation tests (Martin et al., 2017; McDonough, Itrich, et al., 2017). Therefore, the results of screening biodegradation studies, particularly when they suggest a compound is biodegradable, are useful to leverage in a realistic ERA exposure assessment through the inclusion of in‐stream decay (e.g., Federle et al., 2014; McDonough et al., 2016). Considering additional fate processes, for example, sedimentation and photodegradation, as often included in multimedia fate models (Csiszar et al., 2012; Li et al., 2016), would also yield more realistic dissolved water concentrations. However, these processes are not included within the current iSTREEM® framework as it was designed for risk assessment such that concentration estimates are predictive yet conservative. If higher tier exposure estimates would be needed, the addition of further removal fate processes could be investigated (Kapo et al., 2016).

WWTP removal was evaluated via the literature, with data from 10 studies indicating that BP‐3 is well removed during treatment (Table S2). The average removal was 86% across 21 measured values, with 14 of these indicating removal was higher than 86%, which would be expected for a biodegradable chemical. These data were also compared with estimated removals using the SimpleTreat 4.0 WWTP model (Struijs, 2014), which is commonly applied in ERA to estimate removal based on chemical characteristics (EC JRC, 2003, Salvito et al., 2002), as a weight‐of‐evidence approach. The estimated removal by an AS treatment plant is ~71% for BP‐3, that is, with key model inputs of Log *K*_ow_ (3.45) and ready biodegradability (not meeting the 10‐d window). The model predicts that about 63% is biodegraded and 8% sorbs to sludge, with the remainder emitted to surface waters (volatilization is negligible). Experimental WWTP mass balance studies report similar results where BP‐3 losses were determined to be dominantly through biodegradation, 63%, and a smaller fraction due to sorption, 26% (Wang & Kannan, 2017). Although modeled WWTP removal data are lower tier (and thus more conservative) as compared with monitored data, the good agreement between measured and modeled WWTP removal indicates that the measurements being used are reasonable and appropriate.

Further, when chemical properties suggest a compound is expected to be well removed during WWTP treatment *via* biodegradation and/or sorption to sludge, utilizing AS treatment data to populate similar treatment types in iSTREEM® is consistent with findings from the literature. For example, Kasprzyk‐Hordern et al. (2009) studied BP‐3 removal in trickling filter and AS WWTPs and recorded similar removal rates. Leveraging AS removal is ideal because it is the predominant WWTP treatment type in the United States with 86% of wastewater being treated by AS (Kapo et al., 2015), the treatment type most often characterized with experimental removal data (Ramos et al., 2016; Verlicchi et al., 2012), and the treatment type used in WWTP removal models (e.g., SimpleTreat, Struijs, 2014).

#### Comparison of US predicted concentrations with global monitoring data

**Figure 2:**
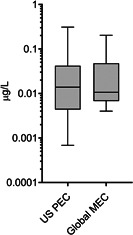
Box plots (5th–95th percentile) of U.S. PECs (*n* = 227 876) and global surface water oxybenzone concentrations (*n* = 42). The top and bottom caps represent the 5th and 95th percentiles, respectively. The line within the boxes represents the median. The bottom and top of the boxes represent the 25th and 75th percentiles, respectively. MEC, measured environmental concentration; PEC, predicted environmental concentration

BP‐3 PECs are compared with the global distribution of concentrations from riverine monitoring campaigns in Figure 2. PECs were compared with global values as an indication that model results are reasonable, in lieu of comparable monitoring data from the United States, as these were lacking in the public domain at the time of the writing of this paper. The global median riverine measured environmental concentration (MEC) was 0.01 µg/L and the 90th percentile MEC was 0.08 µg/L. These values agree with the predicted concentrations, 0.01 and 0.15 µg/L for the 50th and 90th percentile, respectively. More importantly, the range of predicted concentrations closely encompasses the range of measured values.

Global BP‐3 MECs are driven by local variability in both socioeconomic and environmental factors (Kilgallon et al., 2017). Socioeconomic factors including population density, demographics, purchasing power, and wastewater infrastructure influence CPCP usage (Hodges et al., 2014). Meanwhile, environmental factors, particularly flow, also influence observed MECs (Verlicchi et al., 2014), in addition to sampling strategy (Johnson et al., 2008). We were able to identify MECs from riverine environments globally (i.e., 42 data points from 14 countries, Table S6), resulting in the best available BP‐3 MEC distribution, which includes a large variety of socioeconomic and environmental conditions. Notably, much of the monitoring data were collected during warmer months (i.e., during higher usage), which further increases confidence in the assumptions applied in our modeling approach, as model estimates were still conservative as compared with the monitored data. To consider the scenario that daily emissions could be higher in summer months, the effect of emitting all the yearly emissions into half of the year (i.e., by doubling the daily load) was that the modeled concentrations increased by a factor of two. Thus, when comparing with the global MEC, the 90th percentile modeled concentration under the half‐year emission scenario (i.e., 0.3 µg/L) would be more conservative as compared with the global 90th percentile MEC (0.08 µg/L). Given that most measurement data are in summer months and results for the yearly emission were still reasonable and conservative, the approach to assume the consistent yearly emissions for the down‐the‐drain scenario is appropriate.

There are also U.S.‐specific BP‐3 WWTP influent data available to compare our predicted influent concentrations against. Wang and Kannan (2017) observed a median influent concentration of 0.049 µg/L in New York, whereas Loraine and Pettigrove (2006) reported a mean influent concentration of 6.87 µg/L in California. This value is near the second percentile influent PEC calculated in the iSTREEM® modeling, indicating that predicted influent values are greater than those reported for the United States. Though limited, this comparison does provide confidence that our down‐the‐drain emission estimation of BP‐3 is highly conservative. Reed et al. (2017) and Rand et al. (2020) have characterized BP‐3 exposure in Clear Creek, Colorado, over a holiday weekend during significant recreational use, representing a direct discharge scenario. In both studies, the concentration of BP‐3 was below the limit of detection (LOD) upstream of the recreation area and shortly after the recreation ceased (e.g., <0.05​​​​​​ and <0.03 µg/L). This suggests that recreational direct discharge inputs of BP‐3 lead to short‐term concentration pulses rather than long‐term concentration increases. On the basis of their monitoring data, Reed et al. (2017) predicted a recreational BP‐3 release of 0.12 µg/L. Rand et al. (2020) reported short‐term spikes in BP‐3 of 0.05–0.26 µg/L during recreation in 2016. In addition, Rand et al. (2020) conducted a similar monitoring campaign in 2017 that included two other southwest U.S. rivers. The same recreational pulse trend was observed and the 90th percentile MEC was estimated to be 0.68 µg/L. Although the pulse emission data are not directly comparable as they represent short‐term direct discharge emissions, the less than LOD concentrations upstream and after recreation do provide confidence that our down‐the‐drain emission estimation of BP‐3 is realistic as these scenarios are more representative of the scenario modeled.

The MEC comparison presented is not intended to corroborate modeling output, but rather to provide a secondary line of evidence that the PECs are protective, yet reasonable. These results are consistent with conclusions from Kapo et al. (2016) and from Sanderson et al. (2013) who found that the model was consistently conservative when compared with monitoring data for ethoxylate surfactants. These results taken together with previous consumer chemicals exposure assessments with the iSTREEM® model provide strong evidence that the predictions are representative of the U.S. freshwater environment and thus suitable for risk assessment.

### Hazard characterization

The most sensitive aquatic toxicity data and relevant assessment factors used to derive the PNEC are summarized in Table 1. The ECHA registration dossier for BP‐3 contains acute aquatic toxicity data for all three trophic levels, as well as chronic toxicity data for algae (retrieved from ECHA, 2020; Table S4). These studies followed standardized (i.e., OECD) aquatic toxicity test guidelines which are accepted methods in regulatory risk assessments. Additional acute and chronic aquatic toxicity data found in the literature encompassed a variety of relevant lethal and sublethal endpoints, providing a complete and reliable toxicity dataset for all three trophic levels (Table S4). Algae was found to have the most sensitive toxicologically relevant endpoints across both acute and chronic data for the selected taxa and was, therefore, utilized to characterize the environmental hazard of BP‐3. Given that chronic toxicity data were available for all relevant taxa and that the relative sensitivity to BP‐3 across all three trophic levels was less than an order of magnitude in both the acute and chronic exposures, an AF of 10 is appropriate and considered to be protective of the aquatic environment. The resulting freshwater PNEC for BP‐3 was determined to be 18 µg/L, which is similar to the freshwater PNEC of 19.1 µg/L recently published by Carve et al. (2021) utilizing a similar AF‐based method. The PNEC also aligns well with the HC5 (fifth percentile hazardous concentration) of 10.6 µg/L generated by Carve et al. (2021) from a chronic species sensitivity distribution (SSD). A chronic SSD is a valuable higher tier probabilistic tool in environmental risk assessment for evaluating potential hazard to the aquatic environment via a statistical distribution of species‐specific chronic toxicity values (e.g., EC10, NOEC) allowing for generation of a summary toxicity value (i.e., HC5) that is protective of the aquatic community. As the PNEC in this study reflects the currently available aquatic toxicity dataset, it is subject to change upon the availability of new data. However, given that the freshwater aquatic toxicity dataset was relatively complete and sensitivity across trophic levels was similar, it is expected that the freshwater PNEC is unlikely to vary drastically with the availability of new data.

**Table 1:** Summary of the most sensitive acute and chronic aquatic (freshwater) toxicity data by trophic level used in the PNEC derivation

Test type	Trophic level	Concentration (µg/L)	Endpoint	Method	Species	Study Year	Reference
Acute	Algae	670	72 h EC50	OECD 201	*Raphidocelis subcapitata*	2005	ECHA (2020)
	Invertebrate	1100	48 h EC50	GB/T 16125	*Daphnia magna*	2017	Du et al. (2017)
	Fish	2000–4000	6 day LC50	OECD 212	*Danio rerio*	2016	Jang et al. (2016)
Chronic	Algae	**180** a	72 h NOEC	OECD 201	*Raphidocelis subcapitata*	2005	ECHA (2020)
	Invertebrate	≥342	21 day NOEC	OECD 211	*Daphnia magna*	2011	Sieratowicz et al. (2011)
	Fish	191	60 days post hatch NOEC	OECD 235	*Danio rerio*	2015	Kinnberg et al. (2015)
Assessment factor		10					
Freshwater PNEC		18 µg/L					

*Note*: The endpoint used for the final PNEC value in bold. Further details on the reviewed toxicity data can be found in Table S4 and assessment factor guidance in Table S5.

Abbreviations: EC50, median effect concentration; LC50, median lethal concentration; NOEC, no observed effect concentration; PNEC, predicted no‐effect concentration.

^a^ In accordance with regulatory approaches, statistical endpoints from an algal growth inhibition assay can serve as both an acute endpoint (EC50) and chronic endpoint (NOEC/EC10) when chronic data are also present for one other trophic level (ECHA, 2008).

### Environmental risk characterization

A summary of the key iSTREEM® PECs for BP‐3 is presented in Table 2 along with the PNEC. The median predicted exposure concentration is three orders of magnitude less than the PNEC. The disparity between the median PEC and PNEC demonstrates that this level of exposure poses negligible risk. The 75th percentile PEC is also three orders of magnitude less than the PNEC. The 90th percentile predicted exposure concentration is two orders of magnitude lower than the PNEC and for reference, this equates to a low risk quotient, that is, PEC/PNEC less than 0.01, indicating a two order of magnitude margin of safety. In addition, the 90th percentile PEC is double the 90th percentile of globally measured riverine concentrations, further indicating that a conservative PEC was derived using iSTREEM®. In the scenario where BP‐3 loadings were adjusted to occur in just half the year (i.e., double the daily emission), there are still two orders of magnitude between the 90th percentile PEC and PNEC. Therefore, adverse effects from down‐the‐drain release of BP‐3 to receiving riverine environments are considered unlikely. On the basis of this finding of negligible risk, further data collection and refinement within higher tier assessment are not considered high priority for BP‐3 in U.S. freshwaters.

**Table 2:** Summary of iSTREEM® percentiles for BP‐3 exposure in effluent‐receiving rivers in the United States

US PEC percentile (µg/L)				
25th	50th	75th	90th	PNEC (µg/L)
0.004	0.01	0.04	0.15	18

*Note*: PEC percentiles were calculated from the initial concentration per segment in the model, that is, the mixing zone. The PNEC is included as a comparison.

Abbreviations: PEC, predicted environmental concentration; PNEC, predicted no‐effect concentration.

The results of the risk characterization for U.S. freshwater are similar to the conclusions derived by others in previous BP‐3 freshwater ERAs conducted in other regions. Most recently, Carve et al. (2021) compiled a distribution of BP‐3 concentrations measured in rivers and lakes globally. Risk quotients (RQs) were calculated based on the median exposure value and a PNEC of 19.1 µg/L. Negligible risk was identified; however, the authors also calculated a maximal RQ based on their maximal MEC, a clear outlier of 228 µg/L. In this case, the RQ exceeds one, but the relevance of a maximal MEC (which represents a single concentration at a single point in time) to a chronic effect endpoint is limited and not based on standard, accepted ERA approaches (EC JRC, 2003; Nabholz, 1991). In a similar exercise, Guo et al. (2020) collected published freshwater monitoring data from 35 sites globally and also found negligible freshwater risk for BP‐3 based on a reported PNEC of 130 µg/L, which aligns with the conclusion of this study based on the more conservative PNEC of 18 µg/L. Tsui et al. (2014) conducted a monitoring campaign in multiple WWTPs in Hong Kong and divided the highest identified effluent concentration by 10 to estimate environmental exposure. Division by 10 is aligned with EU ERA screening methods (EC JRC, 2003); however, the maximum concentration was used rather than a more representative concentration from the distribution. Furthermore, the PNEC was difficult to interpret, as it was based on in vitro toxicity data. The in vitro test data used in the study have not yet been validated for use in risk assessment, nor were data obtained by following a standardized or modified guideline (e.g., OECD methods). Therefore, conclusions from this risk assessment cannot be reliably understood and instead we have reinterpreted their data using the PNEC derived in Table 1 and estimated the 90th percentile PEC from their data. We determined the 90th percentile PEC (PEC_90_) and maximum PEC (PEC_max_) are not substantially different, that is, 0.04 and 0.05 µg/L, respectively, and thus the PEC_max_ is not a clear outlier in this dataset. Comparing both the PEC_90_ and PEC_max_ with the PNEC yields a result consistent with our findings, with the PECs being two orders of magnitude less than the PNEC, indicating negligible risk.

The ERA method presented in this study is advantageous for several reasons. First, it builds on regulatory guidance relevant to the United States (i.e., Nabholz, 1991) and follows a tiered approach. Tiered ERA approaches are preferable for assessing a large number of chemicals, as lower tiers have minimal data requirements permitting prioritization of chemicals for collection of higher‐tier data for refined exposure assessment. Second, it uses an established spatially explicit exposure modeling approach that requires data inputs that are often readily available for most chemicals. This is a useful tool to conduct reasonable, effective, and proactive distributional analysis of down‐the‐drain exposure nationwide, as iSTREEM® represents spatial variability in freshwater river concentrations at a national scale (Kapo et al., 2016). The toxicity assessment followed a standardized framework that focuses on test species relevant to the region and endpoints deemed suitable for assessing risk, in addition to considering appropriate assessment actors. Taken together, the presented ERA method is a robust framework ideal for assessing the environmental safety of CPCP and OTC ingredients at the national scale.

It should be noted that the goal of the current ERA was to assess the safety of down‐the‐drain releases of BP‐3 to freshwaters, specifically WWTP effluent‐receiving rivers. Although there are BP‐3 monitoring data available from lakes (Table S7), they were not included in our monitoring data distributions (Figure 2A). Lake data are not representative of the environmental compartment modeled nor the emission scenario and are therefore out of scope in this ERA. Specific exposure modeling efforts that incorporate key spatial and temporal hydrologic factors and direct discharge from recreational activities are needed as evidenced by reservoir and lake monitoring (Balmer et al., 2005; O'Malley et al., 2021). Although assessing the safety of direct discharge to near‐shore waters was out of scope of this ERA, we can compare the global distribution of MECs with the freshwater PNEC derived in the study. This preliminary screen indicates that the 90th percentile MEC of BP‐3 in lakes globally was 0.25 µg/L, which is two orders of magnitude below the PNEC (18 µg/L). In addition, the 90th percentile MEC representative of significant recreation in three southwest U.S. rivers was estimated to be 0.68 µg/L, again two orders of magnitude below the PNEC.

## CONCLUSIONS

A freshwater down‐the‐drain risk assessment was conducted for a UV filter of recent regulatory concern, BP‐3, in the conterminous United States. U.S.‐wide surface water concentrations were predicted using the iSTREEM® spatially explicit environmental exposure model. This assessment provides an example of the use of high‐quality emissions data and modeled exposure to assess risk, which is more practical than conducting high‐quality monitoring campaigns for all consumer down‐the‐drain chemicals. The modeled concentrations were compared with the PNEC and risks were determined to be negligible. As risks were not identified based on the available data, it can be concluded that further data collection and refinement within higher tier risk assessment are not necessary for BP‐3 in the freshwater environment. At the time of publication, there were limited U.S. freshwater occurrence data publicly available; however, the range of freshwater river concentrations measured globally falls within the predicted range of concentrations. This paper establishes a framework for down‐the‐drain environmental risk assessments of CPCP and OTC ingredients in the United States.

## CONFLICT OF INTEREST

Emily E. Burns and Ian A. Davies are employees of PCPC. Susan A. Csiszar and Kyle S. Roush are employees of Procter & Gamble Co.

## SUPPORTING INFORMATION

The supplemental data is a single file which includes two figures and seven tables. The figures detail 1) a flow chart outlining freshwater environmental risk assessment (ERA) approach used in this paper, and 2) the locations of the wastewater treatment plants included in the iSTREEM model. The data tables contain reviewed data which supports the analysis conducted in the main text.

## Supporting information

This article contains online‐only Supporting Information.

Supporting information.

## Data Availability

All data and associated metadata not presented in the Supporting Information are available upon request from the corresponding author. The iSTREEM model is a publicly available resource from the American Cleaning Institute via www.istreem.org.
