# Supplementary material for: National scale down‐the‐drain environmental risk assessment of oxybenzone in the United States
Source: Integr Environ Assess Manag. 2021 May 20;17(5):951–60. doi: 10.1002/ieam.4430 (PMC8453704; doi:10.1002/ieam.4430)
Supplement: Supplementary file 1 — Supporting information. [file IEAM-17-951-s001.pdf]

National-scale down-the-drain environmental risk assessment of oxybenzone in the United States

Emily E. Burns[1*], Susan A. Csiszar[2], Kyle Roush[2], and Iain A. Davies[1]

[1] Personal Care Products Council, Washington DC, USA
[2]Procter & Gamble, Cincinnati OH, USA

Supplementary Information

Figure S1. Flow chart outlining freshwater environmental risk assessment (ERA) approach used in this paper. (A) grams/per capita/day (g/c/d); (B) see Table S3 for iSTREEM® input parameters; (C) see Table 2 (main text) for 90th percentile PEC; (D) see Table S4 for identified freshwater toxicity data; (E) see Table 1 (main text) for most sensitive data points and PNEC; (G) See Table S5 for determining the appropriate assessment factor. (F) Refinement of the assessment is required (e.g., collection of more toxicity data or refinement of exposure characterization assumptions) when the PNEC exceeds the PEC. Predicted environmental concentration (PEC); predicted no-effect concentration (PNEC).

Figure S2. The discharge locations of the 13 245 wastewater treatment plants accounted for in the iSTREEM® model.

Table S1. Summary of BP-3 physico-chemical properties.

Table S1. Summary of wastewater treatment removal data from the literature. The mean value across the 22 values in this table was used for the iSTREEM® modeling. The search was limited to activated sludge treatment as this forms the majority of wastewater treatment plants (WWTPs) in the United States.

Table S3: Summary of inputs to iSTREEM® V2.2 to estimate U.S. national mean-flow PECs.

Table S4. Summary of BP-3 aquatic toxicity to standard freshwater test organisms. Hazard characterization completed by application of an appropriate assessment factor to the most sensitive endpoint identified. Only data related to ecologically relevant endpoints (e.g., mortality, growth, reproduction) from the studies conducted according to or similar to standardized test guidelines were selected for use. Greater than values were not considered to be most sensitive when other data was available. Measured values were always selected in preference to nominal values when

available. *Italicized* results were not considered in the assessment. **Bold** results were selected as most sensitive within the trophic level.

Table S5. Summary of assessment factors to derive predicted no-effect concentrations (PNECs) in this study. These assessment factors are based on U.S. Environmental Protection Agency guidance (Nabholz 1991; Zeeman and Gilford, 1993; Beasley et al. 2018)**.**

Table S6: Global monitoring data collected from literature for BP-3 in WWTP effluent and rivers. Data presentation varied by study. In Figure 2 (in the main text) the mean/median was used for distribution calculations. When data was provided for different rivers or WWTPs or at different timepoints, it was not combined into a single value. In certain cases, data needed to be estimated from a graph.[1] This is reported in the 'Notes' column.

Table S7. Global BP-3 monitoring data in lakes or reservoirs collected from the literature. Data presentation varied by study; the mean/median was used in distribution calculations. When data was provided for different locations or at different timepoints, it was not combined into a single value. In certain cases**,** data needed to be estimated from a graph.[1].

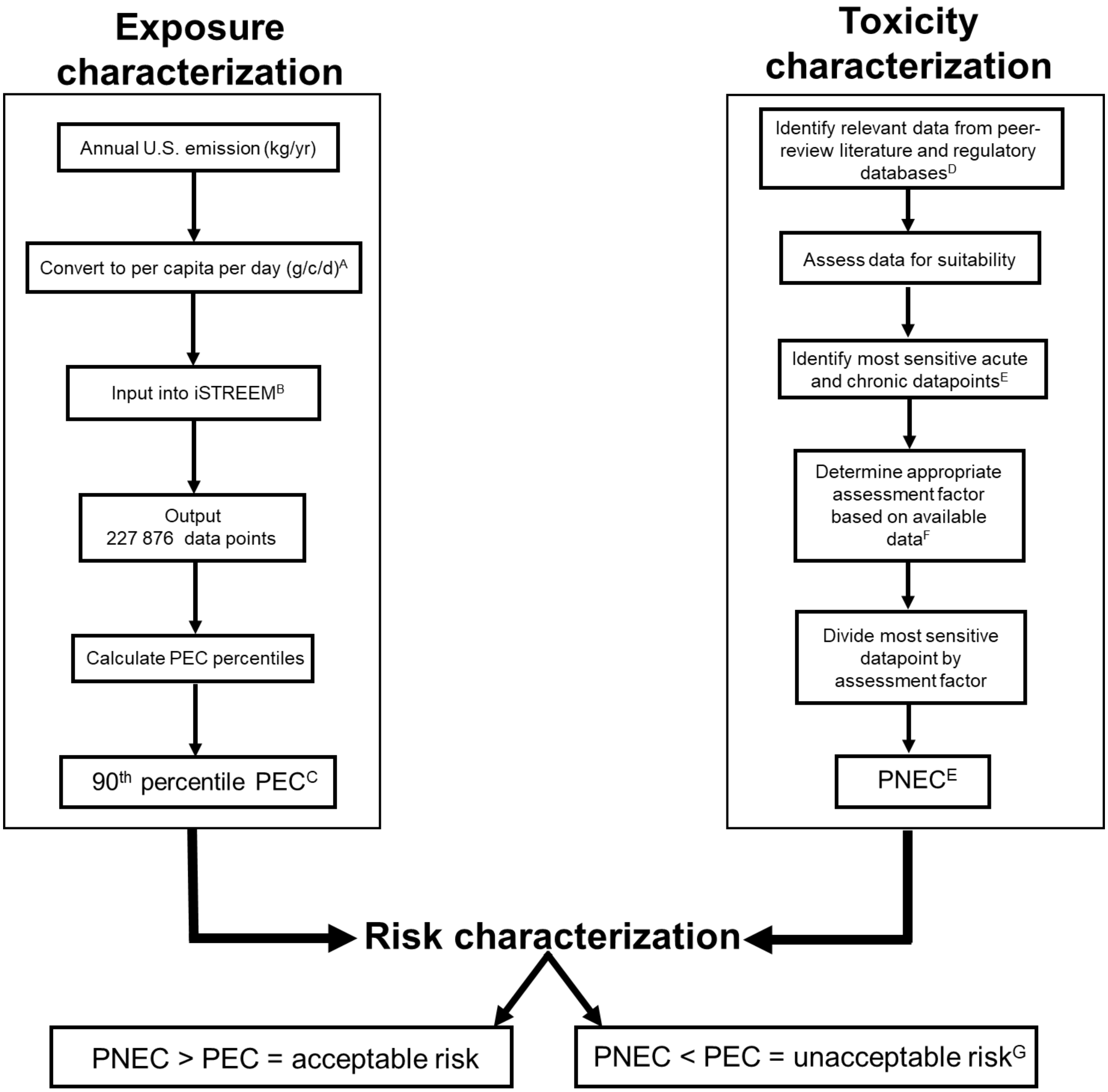

Figure S1. Flow chart outlining freshwater environmental risk assessment (ERA) approach used in this paper. (A) grams/per capita/day (g/c/d); (B) see Table S3 for iSTREEM® input parameters; (C) see Table 2 (main text) for 90th percentile PEC; (D) see Table S4 for identified freshwater toxicity data; (E) see Table 1 (main text) for most sensitive data points and PNEC; (F) See Table S5 for determining the appropriate assessment factor. (G) Refinement of the assessment is required (e.g., collection of more toxicity data or refinement of exposure characterization assumptions) when the PNEC exceeds the PEC. Predicted environmental concentration (PEC); predicted no-effect concentration (PNEC).

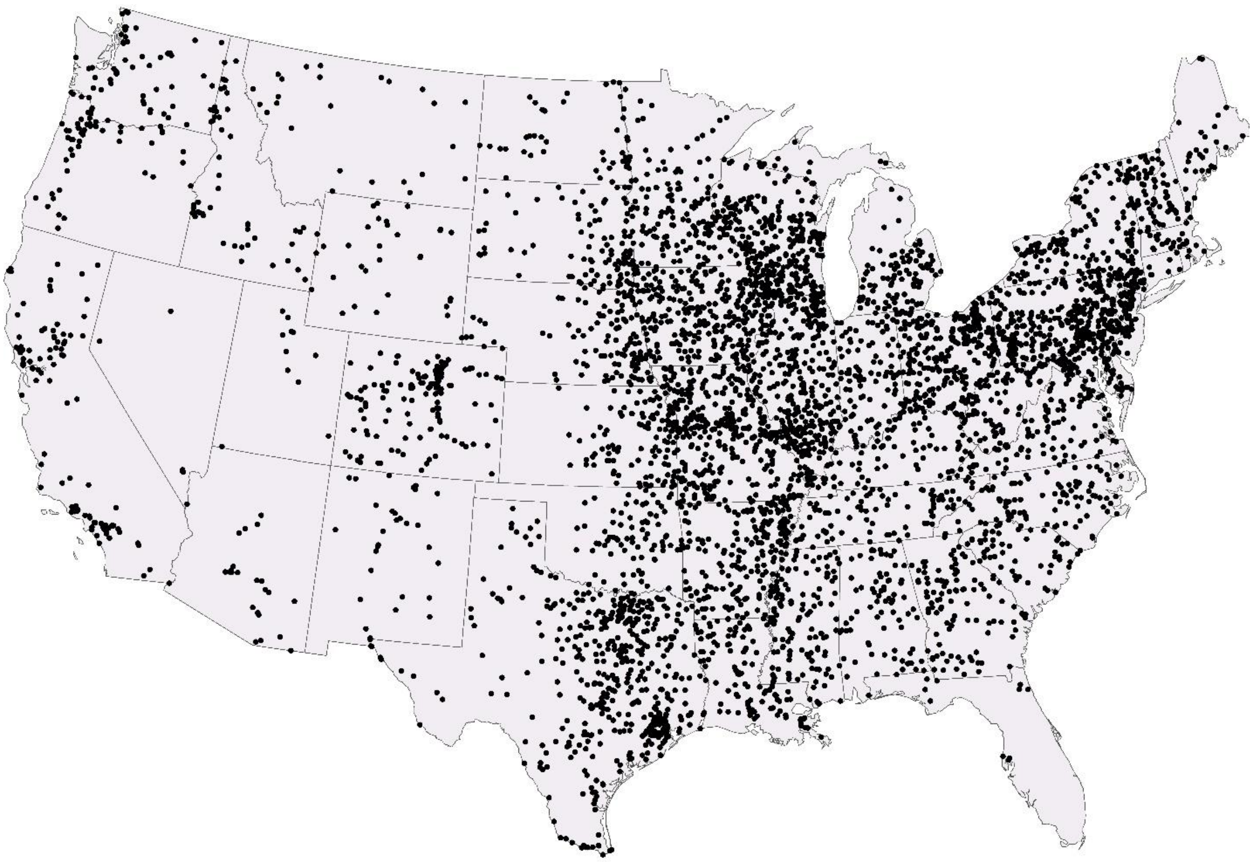

Figure S2. The discharge locations of the 13 245 wastewater treatment plants accounted for in the iSTREEM® model.

Table S1. Summary of oxybenzone (benzophenone-3) physico-chemical properties.

INCI Name (CAS)	Abbreviation	Structure	Solubility[1]	pKa	Log Kow[1]
Benzophenone-3 (131-57-7)	BP-3	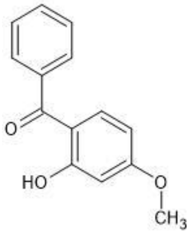	6 mg/L	7.1[2]	3.45

[1]ECHA (2020)
[2]Predicted, National Center for Biotechnology Information (2020)

Table S2. Summary of data for the removal of oxybenzone (BP-3) by wastewater treatment studies published in the literature. The mean value across the 22 values in this table was used for the iSTREEM® modelling. The search was limited to activated sludge treatment as this forms the majority of wastewater treatment plants (WWTPs) in the United States.

**Mean removal (standard deviation)**	**Mean removal value(s) or range reported**	**Reference**
86% (12)	97%[1]	Kasprzyk-Hordern et al. (2009)
	82%[1]	Bueno et al. (2012)
	94%[1] (April), 85%[1] (October)[2]	Liu et al. (2012)
	60% (winter), 62% (summer)	Golovko et al. (2014)
	83% (dry season)[1], 92% (wet season)[1]	Tsui et al. (2014a)
	87%[1]	Ekpeghere et al. (2016)
	86.2%, 91.7%	Wang and Kannan (2017)
	68%, 93%, 93%, 96%, 74%, 99%, 92%, 98%, 99%	Balmer et al. (2005)
	78.2%	Rosal et al. (2010)

[1]Values estimated from a graph using WebPlot Digitizer (Rohatgi 2020).
[2]Removal values reported separately for primary treatment followed by activated sludge. These removals were added together for consistency with other reported values.

Table S3: Summary of inputs to iSTREEM® V2.2 to estimate U.S. national mean-flow PECs.

**Parameter**	**Input**
WWTP removal for: Activated sludge, oxidation ditch, lagoon, trickling filter, and rotating biological contractor	86%
Primary removal	0%
In-stream decay (d^{-1})	0.014 d^{-1}
Emission	0.011 g/c/d

Table S4. Summary of BP-3 aquatic toxicity to standard freshwater test organisms. Hazard characterization completed by application of an appropriate assessment factor to the most sensitive endpoint identified. Only data related to ecologically relevant endpoints (e.g., mortality, growth, reproduction) from the studies conducted according to or similar to standardized test guidelines were selected for use. Greater than values were not considered to be most sensitive when other data was available. Measured values were always selected in preference to nominal values when available. Italicized results were not considered in the assessment. **Bold** results were selected as most sensitive within the trophic level.

	Species	Life stage	Duration	Endpoint	Result (µg/L)	Measured/ Nominal	Guideline	Source	Year	Conclusion
FISH	*Oryzias latipes*	n/a	96 h	LC50	3800	measured	~OECD 203	ECHA	2005	Valid
	Poecilia reticulata	Adult	96 h	LC50	> 1	nominal	n/a	Almeida et al.	2019	Invalid results are non-standard endpoints and/or lack known ecological relevance.
				NOEC (mortality, condition, ertythrotoxicity)	*≥ 1*	nominal				
				NOEC (genotoxicity)	*0.01*	nominal				
				NOEC (genotoxicity)	*0.1*	nominal				
	Danio rerio	n/a	96 h	LC50	3900	n/a	GB/T 27861-2011	Du et al.	2017	Valid
	Danio rerio	Larvae	6 d	*NOEC (gene expression)*	*< 1*	n/a	n/a	Zhang et al.	2017	Non-standard endpoint. Ecological relevance unknown.
	Danio rerio	Eleutheroembryo	48 h	*NOEC (gene expression)*	*100*	measured	n/a	Rodrigues-Fuentes et al.	2015	Non-standard endpoint. Ecological relevance unknown.
	Danio rerio	Embryo	96 hpf	LC50	> 100	nominal	n/a	Tao et al.	2020	Invalid results are non-standard endpoints and/or lack known ecological relevance.
			96 hpf	*NOEC (mortality)*	*≥ 100*	nominal				
		Larvae	96 hpf	*NOEC (neorotoxicity)*	*1*	nominal				
			11 dpf	*NOEC (neurotoxicity)*	*< 10*	nominal				
	Danio rerio	Embryo	96 h	LC50	15900	measured	~OECD 236	Balazs et al.	2016	Valid
			12 0h	LC50	13100	measured				

Table S4. *Continued.*

	Species	Life stage	Duration	Endpoint	Result (µg/L)	Measured/ Nominal	Guideline	Source	Year	Conclusion
FISH	*Danio rerio*	Adult	14 d	LC50	> 312	measured	OECD 204	Bluthgen et al.	2012	Invalid results are non-standard endpoints or exposure and/or lack known ecological relevance.
			14 d	NOEC (mortality, growth, histology)	≥ 312	measured				
			14 d	*NOEC (gene expression)*	*< 2.4*	measured				
		Embryo	120 hpf	LC50	> 438	measured	n/a			
			120 hpf	*NOEC (mortality, behavior)*	*≥ 438*	measured				
			120 hpf	*NOEC (gene expression)*	*8.2*	measured				
	Danio rerio	Embryo (F1)	24 h	*LC50*	*> 550*	nominal	n/a	Li et al.	2018	Invalid results are non-standard endpoints or methods and/or lack known ecological relevance.
			24 h	*NOEC (mortality)*	*5.5*	nominal				
			48 h	*NOEC (heart rate)*	*< 5.5*	nominal				
			72 h	*NOEC (hatch)*	*≥ 550*	nominal				
		Embryo-larvae	48 h	*NOEC (heart rate)*	*< 0.055*	nominal	n/a			
			72 h	*NOEC (hatch)*	*0.055*	nominal				
			7 d	*NOEC (mortality)*	*≥ 550*	nominal				
			7 d	LC50	≥ 550	nominal				
	Danio rerio	Embryo	6 d	**LC50**	**2000 – 4000**	nominal	OECD 212	Jang et al.	2016	Valid
				NOEC (mortality, hatch, defect)	*2000*	nominal				
	Oncorhynchus mykiss	Juvenile	14 d	*NOEC (VTG)*	*80*	measured	n/a	Coronado et al.	2008	Invalid results are non-standard endpoints and/or lack known ecological relevance.
	Oryzias latipes	Adult	21 d	*NOEC (VTG)*	*132*	measured	n/a			
			21 d	NOEC (fecundity)	≥ 620	measured	(~OECD 229)			
			21 d	*NOEC (hatch)*	*132*	measured				

Table S4. *Continued.*

	Species	Life stage	Duration	Endpoint	Result (µg/L)	Measured/ Nominal	Guideline	Source	Year	Conclusion
FISH	*Oryzias latipes*	Adult	14 d	LC50	> 90	measured	n/a	Kim et al.	2014	Invalid results are non-standard endpoints or methods and/or lack known ecological relevance.
			14 d	NOEC (growth, mortality, organ indices)	≥ 90	measured				
			14 d	*NOEC (E2, T, VTG)*	*26*	measured				
			14 d	*NOEC (gene expression)*	*8.4*	measured				
			14 d (28 d total)	*NOEC (reproduction)*	*8.4*	measured				
		Embryo-larvae (F1)	30 dph	*LC50*	*> 500*	nominal				
			30 dph	*NOEC (mortality, hatch, growth)*	*≥ 500*	nominal				
	Pimephales promelas	Juvenile	14 d	LC50	> 3900	measured	n/a	Kunz et al.	2006	Invalid results are non-standard endpoints and/or lack known ecological relevance. Growth endpoint is valid.
			14 d	*NOEC (VTG)*	*≥ 3900*	measured				
			14 d	NOEC (growth)	766	measured				
	Danio rerio	Embryo-Adult	60 dph	*LC50*	*≥ 470*	measured	OECD 234	Kinnberg et al.	2015	Invalid results are non-standard endpoints, lack appropriate conc.-response and/or lack known ecological relevance.
			60 dph	*NOEC (mortality, growth)*	*191*	measured				
			60 dph	**NOEC (sex ratio, gonad maturation)**	**191**	measured				
			60 dph	*NOEC (VTG)*	*≥ 470*	measured				
		Adult	12 d	LC50	> 437	measured	n/a			
			12 d	*NOEC (VTG)*	*63*	measured				

Table S4. *Continued.*

	Species	Life stage	Duration	Endpoint	Result (µg/L)	Measured/ Nominal	Guideline	Source	Year	Conclusion
**FISH**	*Carassius auratus*	n/a	28 d	*LC50*	*> 5*	nominal	n/a	Liu et al.	2015b	Non-standard test species. Invalid results are non-standard endpoints and/or lack known ecological relevance.
			28 d	*NOEC (mortality)*	*> 5*	nominal				
			28 d	*NOEC (hepatic oxidative stress)*	*0.5*	nominal				
	Betta splendens	Adult	28 d	*LC50*	*> 553*	measured	n/a	Chen et al.	2016	Non-standard test species. Invalid results are non-standard endpoints and/or lack known ecological relevance.
			28 d	*NOEC (GSI, mortality)*	*≥ 553*	measured				
			28 d	*NOEC (behavior)*	*< 5.5*	measured				
			28 d	*NOEC (histology)*	*55*	measured				
**INVERTEBRATE**	*Chironomus riparius*	Fourth instar larvae	24 h	LC50	> 10000	nominal	n/a	Ozaez et al.	2013	Invalid results are non-standard endpoints and/or lack known ecological relevance.
			24 h	*NOEC (gene expression, mortality)*	*1000*	nominal				
	Chironomus riparius	Embryo	24 h	*NOEC (gene expression)*	*< 910*	n/a	n/a	Ozaez et al.	2014	Invalid results are non-standard endpoints and/or lack known ecological relevance.
			72 h	*EC50 (hatch)*	*< 910*	n/a				
			72 h	*NOEC (hatch)*	*< 910*	n/a				
			> 72 h (hatch)	LC50	> 910	n/a				
			> 72 h (hatch)	*NOEC (mortality)*	*> 910*	n/a				
	Chironomus riparius	Embryo	24 h	*NOEC (gene expression)*	*< 100*	nominal	n/a	Ozaez et al.	2016	Invalid results are non-standard endpoints and/or lack known ecological relevance.
		Fourth instar larvae	24 h	*NOEC (gene expression)*	*100*	nominal				

Table S4. Continued.

	Species	Life stage	Duration	Endpoint	Result (µg/L)	Measured/ Nominal	Guideline	Source	Year	Conclusion
INVERTEBRATE	*Chironomus riparius*	Fourth instar larvae	8 h	*NOEC (gene expression)*	*< 100*	nominal	n/a	Martin-Folgar et al.	2018	Invalid results are non-standard endpoints and/or lack known ecological relevance.
			24 h	*NOEC (gene expression)*	*< 100*	nominal				
	Chironomus riparius	Fourth instar larvae	8 h	*NOEC (gene expression)*	*< 100*	nominal	n/a	Martinez-Guitarte	2018	Invalid results are non-standard endpoints and/or lack known ecological relevance.
			24 h	*NOEC (gene expression)*	*100*	nominal				
	Chironomus riparius	Fourth instar larvae	8 h	*NOEC (gene expression)*	*< 10*	nominal	n/a	Muniz-Gonzalez and Martinez-Guitarte	2020	Invalid results are non-standard endpoints and/or lack known ecological relevance.
			8 h	*NOEC (enzymatic activity*	*≥ 100*	nominal				
			24 h	*NOEC (gene expression)*	*< 10*	nominal				
			24 h	*NOEC (enzymatic activity)*	*10*	nominal				
			96 h	LC50	> 10000	nominal				
			96 h	*NOEC (mortality)*	*≤ 10*	nominal				
	Daphnia magna	<24h neonate	48 h	EC50 (mobility)	1870	measured	~OECD 202	ECHA	2005	Valid
	Daphnia magna	<24h neonate	48 h	EC50 (mobility)	1900	n/a	OECD 202	Fent et al.	2010	Valid
	Daphnia magna	<8h neonate	48 h	EC50 (mobility)	2170	nominal	OECD 202	Jang et al.	2016	Valid
	Daphnia magna	6-24h neonate	48 h	EC50 (mobility)	2010	n/a	OECD 202	Liu et al.	2015a	Valid
	Daphnia magna	6-24h neonate	48 h	**EC50 (mobility)**	**1100**	nominal	GB/T 16125-2012	Du et al.	2017	Valid
	Daphnia magna	neonate	48 h	EC50 (mobility)	1900	nominal	ISO 6341	Molins-Delgado et al.	2016	Valid

Table S4. Continued.

	Species	Life stage	Duration	Endpoint	Result (µg/L)	Measured/ Nominal	Guideline	Source	Year	Conclusion
INVERTEBRATE	*Dugesia japonica*	n/a	48 h	*LC50*	*900*	nominal	n/a	Li et al.	2012	Non-standard test species without guideline. Additionally, species native to SE Asia and thus not relevant for U.S. ERA.
			96 h	*LC50*	*500*	nominal				
	Daphnia magna	< 24h neonate	48 h	EC50 (mobility)	1200	nominal	~OECD 211	Boyd et al.	2021	Deviations from guideline. Wide and limited exposure concentration selection limits 21d endpoint utility.
			21 d	*NOEC (mortality)*	*2*	nominal				
	Daphnia magna	< 24h neonate	48 h	EC50 (mobility)	1670	nominal	OECD 202	Sieratowicz et al.	2011	Valid
			21 d	**NOEC (mortality, reproduction, growth)**	**≥ 342**	measured	OECD 211			
	Daphnia magna	neonate	24 h	*24h NOEC (feeding)*	*400*	nominal	n/a	Pablos et al.	2015	Invalid endpoint is not standard.
		< 24h neonate	21 d	21d NOEC (mortality, reproduction, growth)	≥ 200	nominal	OECD 211			
ALGAE/	*Raphidocelis subcapitata*	n/a	72 h	**EC50 (growth rate)**	**670**	measured	~OECD 201	ECHA	2005	Valid
			72 h	**NOEC (growth rate)**	**180**	measured				
	Chlorella vulgaris	n/a	72 h	EC50 (growth)	22400	nominal	~OECD 201	Pablos et al.	2015	Valid
			72 h	EC10 (growth)	2200	nominal				

Table S4. Continued.

	Species	Life stage	Duration	Endpoint	Result (µg/L)	Measured/ Nominal	Guideline	Source	Year	Conclusion
**ALGAE/CYANO-BACTERIA**	*Desmodesmus subspicatus*	n/a	72 h	EC50 (growth rate)	960	nominal	OECD 201	Sieratowicz et al.	2011	Valid
			72 h	EC10 (growth rate)	610	nominal				
	Chlamydomonas reinhardtii	n/a	72 h	*EC50 (growth rate)*	*2290*	nominal	OECD 201	Mao et al.	2018	Non-standard test species.
	Chlorella sp	n/a	72 h	*EC50 (dry weight)*	*> 11400*	nominal	n/a	Zhong et al.	2019	Non-standard test species and methods, see Burns and Davies (2020) for further details.
	Arthrospira sp.	n/a	72 h	*NOEC (dry weight)*	*228*	nominal				
			72 h	*EC50 (dry weight)*	*2280 – 11400*	nominal				
			72 h	*NOEC (dry weight)*	*228*	nominal				
	Scenedesmus obliquus	n/a	96h	*EC50 (growth rate)*	*3640*	nominal	n/a	Lee et al.	2020	Non-standard test species without guideline.
	Chlorella vulgaris	n/a	96h	EC50 (growth rate)	2980	nominal	GB/T 21805-2008	Du et al.	2017	Valid
	Chlamydomonas reinhardtii	n/a	96h	*EC50 (growth rate)*	*5000*	nominal	n/a	Esperanza et al.	2019	Non-standard test species without guideline.
			96h	*NOEC (growth rate)*	*1250*	nominal				
	Microcystis aeruginosa	n/a	10d	*EC50 (growth rate)*	*>5000*	nominal	n/a	Mao et al.	2017	Non-standard test species and methods.
	Chlamydomonas reinhardtii	n/a	10d	*EC50 (growth rate)*	*1850*	nominal				

Table S5. Summary of assessment factors to derive predicted no-effect concentrations (PNECs) in this study. These assessment factors are based on U.S. Environmental Protection Agency guidance (Nabholz 1991; Zeeman and Gilford, 1993; Beasley et al. 2018).

Toxicity data available	Assessment factor
Predicted (QSAR)	1000
Acute data (1 or 2 taxa)	1000
Acute data (3 taxa)	100
Chronic data[1] (1 or more taxa)	10
Field study (e.g. microcosm/mesocosm)	1

[1]To apply the chronic data assessment factor (i.e. 10), it must be applied to the most sensitive taxa. The acute toxicity profile can be useful for this purpose.

Table S6. Global monitoring data collected from literature for BP-3 in WWTP effluent and rivers. Data presentation varied by study. In Figure 2 (in the main text) the mean/median was used for distribution calculations. When data was provided for different rivers or WWTPs or at different timepoints, it was not combined into a single value. In certain cases, data needed to be estimated from a graph.[1] This is reported in the 'Notes' column.

	**Matrix (µg/L)**				
**Country**	**Effluent**	**River**	**Result type**	**Notes**	**Reference**
Switzerland	0.3		Mean	Collected at Horgen WWTP in April, 2002.	Balmer et al. (2005)
	0.05		Mean	Collected at Meilen WWTP in April, 2002.	
	0.065		Mean	Collected at Kusnacht WWTP in April, 2002.	
	0.15		Mean	Collected at Männedorf WWTP in April, 2002.	
	0.6		Mean	Collected at Meilen WWTP in June, 2002.	
	0.3		Mean	Collected at Thalwil WWTP in June, 2002.	
	0.7		Mean	Collected at Wädenswil WWTP in June, 2002.	
	0.01		Mean	Collected at Wetzikon WWTP in June, 2003.	
	0.2		Mean	Collected at Männedorf WWTP in Sept., 2003.	
	0.02		Mean	Collected at Thalwil WWTP in Sept., 2003.	
	<0.01		Mean	Collected at Kloten WWTP in Sept., 2003.	
		0.062	Mean, n=2	Samples collected from River Glatt. POCIS were deployed but only results from grab samples were reported. Grab samples collected twice in 2007.	Fent et al. (2010b)
Germany		0.008 (0.007 – 0.011)	Median (range)	Rivers discharging into Baltic Sea. Sampled twice in 2015 at 5 different stations.	Fisch et al. (2017)
	0.431 (0.043)		Mean (SD)	Samples collected for the purpose of method development. Location not provided.	Moeder et al. (2010)
	0.045 (0.005)		Mean (SD)	Collected from WWTP in Leipzig.	Rodil et al. (2009)
	< LOD		Mean (SD)	River Elsterbecken, May 2007.	Rodil and Moeder (2008)
	0.03 (0.003)		Mean (SD)	River Parth, May 2007.	

Table S6. Continued.

Country	Matrix (µg/L)		Result type	Notes	Reference
	Effluent	River			
Germany	0.096 (0.012)		Mean (SD)	WWTP 1	Wick et al. (2010)
	< LOQ		Mean (SD)	WWTP 2; LOQ =25 ng/L.	
		< LOQ	Mean (SD)	River Rhine; LOQ = 5 ng/L.	
		< LOQ	Mean (SD)	Stream 1; LOQ = 5 ng/L.	
		0.047 (0.029)	Mean (SD)	Stream 2.	
Slovenia	0.015 (0.002 – 0.049)	0.007 (0.005 -0.044)	Median (range)	Wastewater treatment plants sampled in Slovenia and Croatia (n=12 samples) The Sava River was sampled at seven locations twice (n=14), including upstream of wastewater discharges.	Česen et al. (2019)
Spain	0.016 (0.008 – 0.034)	n.d. (n.d. – 0.038)	Median (range)	Five river water samples were collected at various points long upper and lower point of Llobregat River in October 2011 Five WWTPs in the Catalonia region sampled October 2011.	Gago-Ferrero et al. (2013)
				Samples were collected from WWTP influent, effluent and surface water (river) from June – September 2008. Samples collected during the highest consumption season (UV filters) with lowest flow rate.	Negreira et al. (2009)
	< LOD	0.054 (0.003)	Mean (SD)	June 2008 sampling. LOD = 13 ng/L.	
	0.083 (0.012)	0.087 (0.008)	Mean (SD)	July 2008 sampling.	
	0.077 (0.004)	< LOD	Mean (SD)	August 2008 sampling. BP-3 LOD = 8 ng/L.	
	0.084 (0.003)	< LOD	Mean (SD)	September 2008 sampling. BP-3 LOD = 8 ng/L.	
				Samples collected in Catalonia.	Pedrouzo et al. (2010)
	n.d.		(n=3)	WWTP A; BP-3 LOD = 5 ng/L.	
	< LOQ		(n=3)	WWTP B; BP-3 LOD = 5 ng/L.	
	< LOQ		(n=3)	WWTP C; BP-3 LOD = 5 ng/L.	
	< LOQ		(n=3)	WWTP D; BP-3 LOD = 5 ng/L.	

Table S6. Continued

Country	Matrix (μg/L)		Result type	Notes	Reference
	Effluent	River			
Spain		0.006	(n=3)	Ebro River	Pedrouzo et al. (2010)
		0.008	(n=3)	Ter River	
		0.028	(n=3)	Llobregat River	
	0.094 (0.048 – 0.14)		Mean (range)	Monitored effluent in 5 WWTPS in Spain over two years. Mean calculated from range.	Bueno et al. (2012)
		0.034 (0.025 – 0.058)	Median (range)	Three samples collected in the Besòs River during May, December and July.	Serra-Roig et al. (2016)
		0.035 (0.002)	Mean (SD)	Samples collected in December 2010. From Guadelete River.	Corada-Fernández et al. (2017)
	0.086 (< LOQ – 0.121)		Mean (range)	Wastewater samples were taken every month from a STP located in Madrid (input and output of the secondary clarifier).	Rosal et al. (2010)
Portugal	0.035 (n.d. – 0.068)		Mean (range)	Values are reported in the text only. Samples collected from 15 different wastewater treatment plants.	Cunha et al. (2015)
Italy				Samples collected from alpine river, Adige River near a tourist area. Values estimated from graph and median taken.	Mandaric et al. (2017)
		0.053 (1.86)	Median (SD)	Summer.	
		0.012 (0.005)	Median (SD)	Winter.	
				Samples collected from 8 municipal wastewater treatment plants in Genoa.	Magi et al. (2013)
	0.005		Mean (SD)	WWTP influent April. Only mean reported for effluent.	
	0.015 (0.008 – 0.028)		Mean (SD)	WWTP influent May.	
	0.008 (0.005 – 0.010)		Mean (SD)	WWTP influent June.	
	0.019 (0.010 – 0.026)		Mean (SD)	WWTP influent July.	
	0.013		Mean (SD)	WWTP influent August. Only mean reported for effluent.	
	0.008 (0.006 – 0.01)		Mean (SD)	WWTP influent September.	

Table S6. Continued.

Country	Matrix (µg/L)		Result type	Notes	Reference
	Effluent	River			
Wales	0.22 (< 0.08 – 0.223)	< 0.015	Mean (range)	Results from River Ely at Talbot Green and WWTP Coslech. LOD reported as 15 ng/L. Note there is a typo in the Table it says µg/L, but refers to ng/L.	Kasprzyk-Hordern et al. (2009)
		< 0.015	Mean	River Ely at Perterson-Super-Ely.	
	0.231 (< 0.08 – 2.2)	0.009 (< 0.015 -0.043)	Mean (range)	Results from River Taff at Abercynon and WWTP Cilfynydd.	
		0.01 (< 0.015 – 0.044)	Mean (range)	River Taff at Pontypridd.	
Norway				Samples collected from two WWTPs in Norway. Daily concentrations were estimated from the graph.	Krzeminski et al. (2017)
	1.10		(n=1)	VEAS Day 1.	
	0.603		(n=1)	VEAS Day 2.	
	0.183		(n=1)	Bekkelaget 1.	
	0.172		(n=1)	Bekkelaget 2.	
	0.143		(n=1)	Bekkelaget 3.	
	0.853 (0.607 -1.10)		Mean (range)	Samples collected from VEAS in WWTP in 2014.	Thomas et al. (2014)
	0.293 (0.081 – 0.598)		Mean (range)	Samples collected from Norway's largest WWTP, VEAS in 2013.	Langford et al. (2015)
	0.233 (0.01 – 0.438)		Mean (range)	Smaller WWTP, HIAS.	
	0.721 (0.374 – 1.92)		Mean (range)	Smaller WWTP Thomasjord.	
USA				Samples collected from two WWTPs in New York state. 24-h composites collected consecutively over a 7-day period.	Wang and Kannan (2017)
	<LOQ (LOQ – 0.012)		Median (SD)	WWTP A, 15 000 population served. LOQ= 0.5 ng/L.	
	<LOQ (LOQ – 0.033)		Median (SD)	WWTP B, 100 000 population served.	

Table S6. Continued.

	Matrix (μg/L)				
**Country**	**Effluent**	**River**	**Result type**	**Notes**	**References**
USA				Samples collected upstream and downstream of recreational activity in Clear Creek at Golden, Colorado. Samples were collected hourly over a 72-h holiday weekend (Labor Day). Based on their data, they predicted a recreational release of 122 ng/L. All values estimated from graphs.	Reed et al. (2017)
		< 0.05 (< 0.05 – 0.09)	Mean (range)	Upstream (n = 72). There was a 1% detection rate.	
		0.07 (< 0.05 – 0.72)	Mean (range)	Downstream (n = 72). There was a 30% detection rate.	
		0.026 – 0.325	Range	Samples collected at three locations in Clear Creek at Golden in Colorado. Mean cannot be calculated as it is unclear how many samples were <LOD. Omitted from MEC distribution	Rand et al. (2020)
				Samples collected from three southwestern rivers over Labor Day weekend, 2017. Samples were collected from an upstream and downstream site at 8 am and 4 pm Sunday – Wednesday.	Rand et al. (2020)
		0.22 (< 0.03 – 0.86)	Mean (range)	Salt River, Arizona.	
		0.06 (< 0.03 – 0.13)	Mean (range)	Truckee River, Nevada.	
		0.25(< 0.03- 0.96)	Mean (range)	Clear Creek, Colorado.	
Australia	0.153 (0.121)		Mean (SD)	Bolivar WWTP in Adelaide, South Australia.	Liu et al. (2012)
	0.033 (0.002)		Mean (SD)	Effluent samples collected from the Bolivar sewage treatment plant in South Australia.	Liu et al. (2011)

Table S6. Continued.

Country	Matrix (µg/L)		Result type	Notes	References
	Effluent	River			
Australia				Samples collected in August 2011 from four rivers that drain into Port Philip Bay near Melbourne. Only the mean concentration is provided.	Allinson et al. (2018)
		0.006	Mean	Mouth of Werribee River.	
		0.004	Mean	Mouth of Kororit Creek.	
		0.007	Mean	Yarra River estuary.	
		0.004	Mean	Maribyrnong River prior to confluence with Yarra River.	
China				Samples collected at the influent and effluent of the Tianjin wastewater treatment plant. Includes ozonation as the secondary treatment.	Li et al. (2007)
	0.086 (0.068 -0.103)		Mean (range)	Collected February 2005.	
	0.438 (0.377 – 0.506)		Mean (range)	Collected July 2005.	
	0.226 (0.210 -0.246)		Mean (range)	Collected September 2005.	
		0.010 (0.03)	Mean (max.)	Samples (n=27) were collected along the Huangpu River and the surface water in and near Water Park within Shanghai.	Wu et al. (2017)
Hong Kong	0.111 (0.541)		Mean (SD)	Samples were collected from 5 WWTP equipped with different treatment levels.	Tsui et al. (2014a)
Taiwan				Two municipal wastewater treatment plants, effluents were collected in different months in the city of Tainan. Surface water samples came from two major rivers in northern Taiwan (where the effluent would be discharged).	Wu et al. (2013)
	0.021		(n=1)	WWTP Effluent 1.	
	0.013		(n=1)	WWTP Effluent 2.	
		0.012	(n=1)	River Water 1.	
		0.015	(n=1)	River Water 2.	

Table S6. Continued.

Country	Matrix (µg/L) Effluent	River	Result type	Notes	References
Japan				Twenty-nine sampling sites in rivers of various anthropogenic influence.	Kameda et al. (2011)
		0.025 (0.016 – 0.041)	Mean (range)	Effluent fed stream, 2 sampling sites.	
		0.004	(n=1)	Heavily polluted rivers, 6 sampling sites.	
		0.006 (0.004 – 0.012)	Mean (range)	Moderately polluted rivers, 12 sampling sites.	
		0.004 (0.002 – 0.01)	Mean (range)	Background river, 5 sampling sites.	
	0.054 (0.029 – 0.164)		Mean (range)	WWTP effluents, four sampling points.	
South Korea	0.143 (<LOD – 0.502)	0.009 (<LOD – 0.156)	Median (range)	Four wastewater treatment plants sampled. Surface water samples were collected from eleven sites along three South Korean rivers. Median concentrations were estimated from graphs. For consistency, sewage treatment data not included.	Ekpeghere et al. (2016)
Thailand		0.101 (0.086 -0.116)	Median (range)	Surface water samples were collected from 8 locations; however, these were all marine surface water with the exception of the Bangkok samples (n=2).	Tsui et al. (2014b)
Singapore				Tropical urban watershed. The main water basin receives discharge from 5 major tributaries.	Mao et al. (2018)
		0.008 (0.002 -1.23)	Median (range)	Tributary.	
				Tropical urban watershed. The main water reservoir receives discharge from 5 major tributaries.	You et al. (2015)
		0.011 (<0.001 – 0.224)	Median (range)	Tributaries.	

[1]Values estimated from a graph using WebPlot Digitizer (Rohatgi 2020). Not detected = n.d.; maximum = max.; limit of detection = LOD; limit of quantification = LOQ.

Table S7. Global BP-3 monitoring data in lakes or reservoirs collected from the literature. Data presentation varied by study; the mean/median was used in distribution calculations. When data was provided for different locations or at different timepoints, it was not combined into a single value. In certain cases**,** data needed to be estimated from a graph.[1]

Country	Lake water (µg/L)	Result type	Notes	Reference
Switzerland	0.016	Mean (n=2)	Collected from Lake Zurichsee, August 2002.	Balmer et al. (2005)
	0.011	Mean (n=3)	Collected from Lake Zurichsee, September 2002.	
	0.020	(n=1)	Collected from Lake Grifensee, August 2002.	
	0.030	(n=1)	Collected from Lake Grifensee, July 2002.	
	0.035	(n=1)	Collected from Lake Huttnersee, July 8, 2002.	
	0.023	(n=1)	Collected from Lake Huttnersee, July 26, 2002.	
Germany	0.083 (0.011)	Mean (SD)	Samples collected from Germany surface water for the purpose of method development. Location not provided.	Moeder et al. (2010)
	0.04 (0.003)	Mean (SD)	Surface water was collected at the Lake Cospuden.	Rodil et al. (2009)
	<LOD	Mean (SD)	Lake Cospudener, May 2007. LOD = 11 ng/L.	Rodil and Moeder (2008)
	0.027 (0.004)	Mean (SD)	Lake Cospudener, June 2007.	
	0.017 (0.002)	Mean (SD)	Lake Bagger, May 2007.	
	0.055 (0.011)	Mean (SD)	Lake Bagger, June 2007.	
Australia	0.646 (0.352 – 0.888)	Median (R)	Samples collected at various time points (12 h) from a freshwater reservoir during swimming. Data estimated from graph.	O'Malley et al. (2021)
France	0.003	Median	Sample collected from Villeneuve-de-la-Raho (artificial lake). This site sees highly increased tourism pressure during the summer months.	Fagervold et al. (2019)
Singapore	0.01 (0.002 – 0.331)	Median (R)	Tropical urban watershed. The main water reservoir receives discharge from 5 major tributaries.	You et al. (2015)
	0.007 (0.005 - 0.056)	Median (R)	Same basin (reservoir) sampled as You et al. (2015).	Mao at al. (2018)

[1]Values estimated from a graph using WebPlot Digitizer (Rohatgi 2020).

LITERATURE CITED

Allinson M, Kameda Y, Kimura K, Allinson G. 2018. Occurrence and assessment of the risk of ultraviolet filters and light stabilizers in Victorian estuaries. *Environ Sci Pollut Res*. 25(12):12022–12033. doi:10.1007/s11356-018-1386-7.

Almeida S, Rocha T, Qualhato G, Oliveira L, Amaral C, Conceiçcao E, Saboia-Morais S, Bailao E. 2019. Acute exposure to environmentally relevant concentrations of benzophenone-3 induced genotoxicity in *Poecilia reticulata*. *Aquat. Toxicol.* 216:105293. https://doi.org/10.1016/j.aquatox.2019.105293.

Balázs A, Krifaton C, Orosz I, Szoboszlay S, Kovács R, Csenki Z, Urbányi B, Kriszt B. 2016. Hormonal activity, cytotoxicity and developmental toxicity of UV filters. *Ecotoxicol Environ Saf*. 131:45–53. doi:10.1016/j.ecoenv.2016.04.037.

Balmer ME, Buser HR, Müller MD, Poiger T. 2005. Occurrence of some organic UV filters in wastewater, in surface waters, and in fish from Swiss lakes. *Environ Sci Technol.* 39(4):953–962. doi:10.1021/es040055r.

Beasley A, Belanger S, Connors KA, Embry MR, Otter R. 2018. EnviroTox Platform: EnviroTox database, PNEC calculator, ecoTTC, Chemical toxicity distribution (CTD). User Guide. Washington (DC): Health and Environmental Sciences Institute [accessed 2020/12/07] https://envirotoxdatabase.org/index.php/documentation.

Blüthgen N, Zucchi S, Fent K. 2012. Effects of the UV filter benzophenone-3 (oxybenzone) at low concentrations in zebrafish (*Danio rerio*). *Toxicol Appl Pharmacol*. 263(2):184–194. doi:10.1016/j.taap.2012.06.008.

Boyd A, Stewart CB, Philibert DA, How ZT, El-Din MG, Tierney KB, Blewett TA. 2021. A burning issue: The effect of organic ultraviolet filter exposure on the behaviour and physiology of *Daphnia magna*. *Sci Total Environ.* 750:141707. doi:10.1016/j.scitotenv.2020.141707.

Bueno MJM, Gomez MJ, Herrera S, Hernando MD, Agüera A, Fernández-Alba AR. 2012. Occurrence and persistence of organic emerging contaminants and priority pollutants in five sewage treatment plants of Spain: Two years pilot survey monitoring. *Environ Pollut*. 164:267–273. doi:10.1016/j.envpol.2012.01.038.

Burns EE, Davies IA. 2020. The toxicological effects of oxybenzone, an active ingredient in sunscream personal care products, on prokaryotic alga *Arthrospira* sp. and eukaryotic algae *Chlorella* sp.: Methodological issues. *Aquat. Toxicol.* 226:105501.

Česen M, Ahel M, Terzić S, Heath DJ, Heath E. 2019. The occurrence of contaminants of emerging concern in Slovenian and Croatian wastewaters and receiving Sava river. *Sci Total Environ*. 650:2446–2453. doi:10.1016/j.scitotenv.2018.09.238.

Chen, TH, Wu YT, Ding WH. 2016. UV-filter benzophenone-3 inhibits agonistic behavior in male Siamese fighting fish (*Betta splendens*). *Ecotoxicology*. 25:302-309. https://doi.org/10.1007/s10646-015-1588-4.

Corada-Fernández C, Candela L, Torres-Fuentes N, Pintado-Herrera MG, Paniw M, González-Mazo E. 2017. Effects of extreme rainfall events on the distribution of selected emerging

contaminants in surface and groundwater: The Guadalete River basin (SW, Spain). *Sci Total Environ*. 605–606:770–783. doi:10.1016/j.scitotenv.2017.06.049.

Coronado M, De Haro H, Deng X, Rempel MA, Lavado R, Schlenk D. 2008. Estrogenic activity and reproductive effects of the UV-filter oxybenzone (2-hydroxy-4-methoxyphenyl-methanone) in fish. *Aquat Toxicol*. 90(3):182–187. doi:10.1016/j.aquatox.2008.08.018.

Cunha SC, Pena A, Fernandes JO. 2015. Dispersive liquid-liquid microextraction followed by microwave-assisted silylation and gas chromatography-mass spectrometry analysis for simultaneous trace quantification of bisphenol A and 13 ultraviolet filters in wastewaters. *J Chromatogr A*. 1414:10–21. doi:10.1016/j.chroma.2015.07.099.

Du Y, Wang WQ, Pei ZT, Ahmad F, Xu RR, Zhang YM, Sun LW. 2017. Acute toxicity and ecological risk assessment of benzophenone-3 (BP-3) and benzophenone-4 (BP-4) in ultraviolet (UV)-filters. *Int J Environ Res Public Health.* 14(11):1414. doi:10.3390/ijerph14111414.

[ECHA] European Chemicals Agency. 2020. Information on chemicals: registration dossier, oxybenzone. [cited 2020/12/07] https://echa.europa.eu/registration-dossier/-/registered-dossier/5515/1.

Ekpeghere KI, Kim U-J, O SH, Kim H-Y, Oh J-E. 2016. Distribution and seasonal occurrence of UV filters in rivers and wastewater treatment plants in Korea. *Sci Total Environ.* 542:121–128. doi:10.1016/j.scitotenv.2015.10.033.

Esperanza M, Seoane M, Rioboo C, Herrero C, Cid Á. 2019. Differential toxicity of the UV-filters BP-3 and BP-4 in *Chlamydomonas reinhardtii*: A flow cytometric approach. *Sci Total Environ.* 669:412–420. doi:10.1016/j.scitotenv.2019.03.116.

Fagervold SK, Rodrigues AS, Rohée C, Roe R, Bourrain M, Stien D, Lebaron P. 2019. Occurrence and Environmental Distribution of 5 UV Filters During the Summer Season in Different Water Bodies. *Water Air Soil Pollut.* 230(7). doi:10.1007/s11270-019-4217-7.

Fent K, Kunz PY, Zenker A, Rapp M. 2010a. A tentative environmental risk assessment of the UV-filters 3-(4-methylbenzylidene-camphor), 2-ethyl-hexyl-4-trimethoxycinnamate, benzophenone-3, benzophenone-4 and 3-benzylidene camphor. *Mar Environ Res.* 69:S4–S6. doi:10.1016/j.marenvres.2009.10.010.

Fent K, Zenker A, Rapp M. 2010b. Widespread occurrence of estrogenic UV-filters in aquatic ecosystems in Switzerland. *Environ Pollut.* 158(5):1817–1824. doi:10.1016/j.envpol.2009.11.005.

Fisch K, Waniek JJ, Schulz-Bull DE. 2017. Occurrence of pharmaceuticals and UV-filters in riverine run-offs and waters of the German Baltic Sea. *Mar Pollut Bull.* 124(1):388–399. doi:10.1016/j.marpolbul.2017.07.057.

Gago-Ferrero P, Mastroianni N, Díaz-Cruz MS, Barceló D. 2013. Fully automated determination of nine ultraviolet filters and transformation products in natural waters and wastewaters by on-line solid phase extraction-liquid chromatography-tandem mass spectrometry. *J Chromatogr A.* 1294:106–116. doi:10.1016/j.chroma.2013.04.037.

Golovko O, Kumar V, Fedorova G, Randak T, Grabic R. 2014. Removal and seasonal variability

of selected analgesics/anti-inflammatory, anti-hypertensive/cardiovascular pharmaceuticals and UV filters in wastewater treatment plant. *Environ Sci Pollut Res.* 21(12):7578–7585. doi:10.1007/s11356-014-2654-9.

Jang GH, Park CB, Kang BJ, Kim YJ, Lee KH. 2016. Sequential assessment via daphnia and zebrafish for systematic toxicity screening of heterogeneous substances. *Environ Pollut.* 216:292–303. doi:10.1016/j.envpol.2016.06.001.

Kameda Y, Kimura K, Miyazaki M. 2011. Occurrence and profiles of organic sun-blocking agents in surface waters and sediments in Japanese rivers and lakes. *Environ Pollut.* 159(6):1570–1576. doi:10.1016/j.envpol.2011.02.055.

Kasprzyk-Hordern B, Dinsdale RM, Guwy AJ. 2009. The removal of pharmaceuticals, personal care products, endocrine disruptors and illicit drugs during wastewater treatment and its impact on the quality of receiving waters. *Water Res*. 43:363–380. doi:10.1016/j.watres.2008.10.047.

Kim S, Jung D, Kho Y, Choi K. 2014. Effects of benzophenone-3 exposure on endocrine disruption and reproduction of japanese medaka (*Oryzias latipes*)-A two generation exposure study. *Aquat Toxicol*. 155:244–252. doi:10.1016/j.aquatox.2014.07.004.

Kinnberg KL, Petersen GI, Albrektsen M, Minghlani M, Awad SM, Holbech BF, Green JW, Bjerregaard P, Holbech H. 2015. Endocrine-disrupting effect of the ultraviolet filter benzophenone-3 in zebrafish, *Danio rerio. Environ Toxicol Chem.* 34(12):2833–2840. doi:10.1002/etc.3129.

Krzeminski P, Schwermer C, Wennberg A, Langford K, Vogelsang C. 2017. Occurrence of UV filters, fragrances and organophosphate flame retardants in municipal WWTP effluents and their removal during membrane post-treatment. *J Hazard Mater.* 323:166–176. doi:10.1016/j.jhazmat.2016.08.001.

Kunz P, Galicia H, Fent K. 2006. Comparison of in vitro and in vivo estrogenic activity of UV filters in fish. *Toxicol Sci*. 90:349-361. https://doi.org/10.1093/toxsci/kfj082.

Langford KH, Reid MJ, Fjeld E, Øxnevad S, Thomas K V. 2015. Environmental occurrence and risk of organic UV filters and stabilizers in multiple matrices in Norway. *Environ Int.* 80:1–7. doi:10.1016/j.envint.2015.03.012.

Lee SH, Xiong JQ, Ru S, Patil SM, Kurade MB, Govindwar SP, Oh SE, Jeon BH. 2020. Toxicity of benzophenone-3 and its biodegradation in a freshwater microalga *Scenedesmus obliquus*. *J Hazard Mater*. 389:122149. doi:10.1016/j.jhazmat.2020.122149.

Li AJ, Law JC-FF, Chow C-HH, Huang Y, Li K, Leung KS-YY 2018. Joint effects of multiple UV filters on zebrafish embryo development. *Environ Sci Technol*. 52:9460-9467. https://doi.org/10.1021/acs.est.8b02418.

Li MH. 2012. Acute toxicity of benzophenone-type UV filters and paraben preservatives to freshwater planarian, *Dugesia japonica*. *Toxicol Environ Chem*. 94(3):566–573. doi:10.1080/02772248.2012.655695.

Li W, Ma Y, Guo C, Hu W, Liu K, Wang Y, Zhu T. 2007. Occurrence and behavior of four of the

most used sunscreen UV filters in a wastewater reclamation plant. *Water Res.* 41(15):3506–3512. doi:10.1016/j.watres.2007.05.039.

Liu H, Sun P, Liu H, Yang S, Wang L, Wang Z. 2015a. Acute toxicity of benzophenone-type UV filters for *Photobacterium phosphoreum* and *Daphnia magna*: QSAR analysis, interspecies relationship and integrated assessment. *Chemosphere.* 135:182–188. doi:10.1016/j.chemosphere.2015.04.036.

Liu H, Sun P, Yang S, Wang L,Wang Z. 2015b. Hepatic oxidative stress biomarker responses in freshwater fish *Carassius auratus* exposed to four benzophenone UV filters. *Ecotoxicol Environ Saf.* 119:116-122. https://doi.org/10.1016/j.ecoenv.2015.05.017.

Liu YS, Ying GG, Shareef A, Kookana RS. 2011. Simultaneous determination of benzotriazoles and ultraviolet filters in ground water, effluent and biosolid samples using gas chromatography-tandem mass spectrometry. *J Chromatogr A.* 1218(31):5328–5335. doi:10.1016/j.chroma.2011.05.100.

Liu YS, Ying GG, Shareef A, Kookana RS. 2012. Occurrence and removal of benzotriazoles and ultraviolet filters in a municipal wastewater treatment plant. *Environ Pollut.* 165:225–232. doi:10.1016/j.envpol.2011.10.009.

Magi E, Scapolla C, Di Carro M, Rivaro P, Ngoc Nguyen KT. 2013. Emerging pollutants in aquatic environments: Monitoring of UV filters in urban wastewater treatment plants. *Anal Methods.* 5(2):428–433. doi:10.1039/c2ay26163d.

Mandaric L, Diamantini E, Stella E, Cano-Paoli K, Valle-Sistac J, Molins-Delgado D, Bellin A, Chiogna G, Majone B, Diaz-Cruz MS, Sabater S, Barcelo D, Petrovic M. 2017. Contamination sources and distribution patterns of pharmaceuticals and personal care products in Alpine rivers strongly affected by tourism. *Sci Total Environ.* 590–591:484–494. doi:10.1016/j.scitotenv.2017.02.185.

Mao F, He Y, Kushmaro A, Gin KYH. 2017. Effects of benzophenone-3 on the green alga *Chlamydomonas reinhardtii* and the cyanobacterium *Microcystis aeruginosa*. *Aquat Toxicol.* 193:1–8. doi:10.1016/j.aquatox.2017.09.029.

Mao, F., He, Y., Gin, K.-H.K.Y.H. 2018. Evaluating the joint toxicity of two benzophenone-type UV filters on the green alga *Chlamydomonas reinhardtii* with response surface methodology. *Toxics.* 6:1-12. https://doi.org/10.3390/ toxics6010008.

Mao F, You L, Reinhard M, He Y, Gin KYH. 2018. Occurrence and Fate of Benzophenone-Type UV Filters in a Tropical Urban Watershed. *Environ Sci Technol.* 52(7):3960–3967. doi:10.1021/acs.est.7b05634.

Martín-Folgar R, Aquilino M, Ozaez I, Martínez-Guitarte JL. 2018. Ultraviolet filters and heat shock proteins: effects in *Chironomus riparius* by benzophenone-3 and 4-methylbenzylidene camphor. *Environ Sci Pollut*. Res. 25:333-344. https://doi.org/10.1007/s11356-017-0416-1.

Martinez-Guitarte JL. 2018. Transcriptional activity of detoxification genes is altered by ultraviolet filters in *Chironimus riparius*. *Ecotox Environ Saf.* 149:64-71. https://doi.org/10.1016/j.ecoenv.2017.11.017.

Moeder M, Schrader S, Winkler U, Rodil R. 2010. At-line microextraction by packed sorbent-gas chromatography-mass spectrometry for the determination of UV filter and polycyclic musk compounds in water samples. *J Chromatogr A*. 1217(17):2925–2932. doi:10.1016/j.chroma.2010.02.057.

Molins-Delgado D, Gago-Ferrero P, Díaz-Cruz MS, Barceló D. 2016. Single and joint ecotoxicity data estimation of organic UV filters and nanomaterials toward selected aquatic organisms. Urban groundwater risk assessment. *Environ Res*. 145:126–134. doi:10.1016/j.envres.2015.11.026.

Muniz-Gonzalez A-B, Martínez-Guitarte J-L. 2020. Combined effects of benzophenone-3 and temperature on gene expression and enzymatic activity in the aquatic larvae *Chironomus riparius*. *Sci Total Environ*. 698:134292. https://doi.org/10.1016/j.scitotenv.2019.134292.

Nabholz J V. 1991. Environmental hazard and risk assessment under the United States Toxic Substances Control Act. *Sci Total Environ.* 109–110:649–665. doi:10.1016/0048-9697(91)90218-4.

National Center for Biotechnology Information (2020). PubChem Compound Summary for CID 4632, Oxybenzone. [Accessed 2020/12/08] https://pubchem.ncbi.nlm.nih.gov/compound/Oxybenzone.

Negreira N, Rodríguez I, Ramil M, Rubí E, Cela R. 2009. Solid-phase extraction followed by liquid chromatography-tandem mass spectrometry for the determination of hydroxylated benzophenone UV absorbers in environmental water samples. *Anal Chim Acta.* 654(2):162–170. doi:10.1016/j.aca.2009.09.033.

O’Malley E, McLachlan MS, O’Brien JW, Verhagen R, Mueller JF. 2021. The presence of selected UV filters in a freshwater recreational reservoir and fate in controlled experiments. *Sci Total Environ.* 754:142373. doi:10.1016/j.scitotenv.2020.142373.

Ozaez I, Aquilino M, Morcillo G, Martínez-Guitarte JL. 2016. UV filters induce transcriptional changes of different hormonal receptors in *Chironomus riparius* embryos and larvae. *Environ Pollut*. 214:239-247. http://dx.doi.org/10.1016/j.envpol.2016.04.023.

Ozaez I, Martínez-Guitarte JL, Morcillo G. 2014. The UV filter benzophenone 3 (BP-3) activates hormonal genes mimicking the action of ecdysone and alters embryo development in the insect *Chironomus riparius* (Diptera). *Environ Pollut*. 192:19-26. https://doi.org/10.1016/j.envpol.2014.04.038.

Ozaez I, Martínez-Guitarte JL, Morcillo G. 2013. Effects of in vivo exposure to UV filters (4-MBC, OMC, BP-3, 4-HB, OC, OD-PABA) on endocrine signaling genes in the insect *Chironomus riparius*. *Sci Total Environ*. 456-457:120-126. https://doi.org/10.1016/j.scitotenv.2013.03.081.

Pablos MV, García-Hortigüela P, Fernández C. 2015. Acute and chronic toxicity of emerging contaminants, alone or in combination, in *Chlorella vulgaris* and *Daphnia magna*. Environ Sci Pollut Res. 22:5417–5424. doi:10.1007/s11356-015-4119-1.

Pedrouzo M, Borrull F, Marcé RM, Pocurull E. 2010. Stir-bar-sorptive extraction and ultra-high-performance liquid chromatography-tandem mass spectrometry for simultaneous analysis of

UV filters and antimicrobial agents in water samples. *Anal Bioanal Chem.* 397:2833–2839. doi:10.1007/s00216-010-3743-3.

Rand LN, Bi Y, Poustie A, Bednar AJ, Hanigan DJ, Westerhoff P, Ranville JF. 2020. Quantifying temporal and geographic variation in sunscreen and mineralogic titanium-containing nanoparticles in three recreational rivers. *Sci Total Environ.* 743:140845. doi:10.1016/j.scitotenv.2020.140845.

Reed RB, Martin DP, Bednar AJ, Montaño MD, Westerhoff P, Ranville JF. 2017. Multi-day diurnal measurements of Ti-containing nanoparticle and organic sunscreen chemical release during recreational use of a natural surface water. *Environ Sci Nano.* 4(1):69–77. doi:10.1039/c6en00283h.

Rodil R, Moeder M. 2008. Development of a method for the determination of UV filters in water samples using stir bar sorptive extraction and thermal desorption-gas chromatography-mass spectrometry. *J Chromatogr A.* 1179(2):81–88. doi:10.1016/j.chroma.2007.11.090.

Rodil R, Schrader S, Moeder M. 2009. Non-porous membrane-assisted liquid-liquid extraction of UV filter compounds from water samples. *J Chromatogr A.* 1216(24):4887–4894. doi:10.1016/j.chroma.2009.04.042.

Rodríguez-Fuentes G, Sandoval-Gío JJ, Arroyo-Silva A, Noreña-Barroso E, Escalante-Herrera KS, Olvera-Espinosa F. 2015. Evaluation of the estrogenic and oxidative stress effects of the UV filter 3-benzophenone in zebrafish (*Danio rerio*) eleuthero-embryos. *Ecotoxicol Environ Saf*. 115:14–18. doi:10.1016/j.ecoenv.2015.01.033.

Rohatgi A. 2020. WebPlotDigitizer, Version 4.3. Pacifica, CA, USA. [accessed Nov. 12]. https://automeris.io/WebPlotDigitizer

Rosal R, Rodríguez A, Perdigón-Melón JA, Petre A, García-Calvo E, Gómez MJ, Agüera A, Fernández-Alba AR. 2010. Occurrence of emerging pollutants in urban wastewater and their removal through biological treatment followed by ozonation. *Water Res.* 44(2):578–588. doi:10.1016/j.watres.2009.07.004.

Serra-Roig MP, Jurado A, Díaz-Cruz MS, Vázquez-Suñé E, Pujades E, Barceló D. 2016. Occurrence, fate and risk assessment of personal care products in river–groundwater interface. *Sci Total Environ*. 568:829–837. doi:10.1016/j.scitotenv.2016.06.006.

Sieratowicz A, Kaiser D, Behr M, Oetken M, Oehlmann J. 2011. Acute and chronic toxicity of four frequently used UV filter substances for *Desmodesmus subspicatus* and *Daphnia magna*. *J Environ Sci Heal - Part A Toxic/Hazardous Subst Environ Eng.* 46(12):1311–1319. doi:10.1080/10934529.2011.602936.

Tao J, Bai C, Chen Y, Zhou H, Liu Y, Shi Q, Pan W, Dong H, Li L, Xu H, Tanguay R, Huang C, Dong Q. 2020. Environmental relevant concentrations of benzophenone-3 induced developmental neurotoxicity in zebrafish. *Sci Total Environ*. 721:137686. https://doi.org/10.1016/j.scitotenv.2020.137686.

Thomas K, Schlabach M, Langford K, Fjeld E, Øxnevad S, Rundberget T, Bæk K, Rostkowski P, Harju M. 2014. Screening programme 2013: New bisphenols, organic peroxides, fluorinated

siloxanes, organic UV filters and selected PBT substances. Oslo (NO): Norwegian Environment Agency. Contract Report M-176/2014.

Tsui Mirabelle M P, Leung HW, Lam PKS, Murphy MB. 2014a. Seasonal occurrence, removal efficiencies and preliminary risk assessment of multiple classes of organic UV filters in wastewater treatment plants. *Water Res.* 53:58–67. doi:10.1016/j.watres.2014.01.014.

Tsui Mirabelle M.P., Leung HW, Wai TC, Yamashita N, Taniyasu S, Liu W, Lam PKS, Murphy MB. 2014b. Occurrence, distribution and ecological risk assessment of multiple classes of UV filters in surface waters from different countries. *Water Res.* 67:55–65. doi:10.1016/j.watres.2014.09.013.

Wang W, Kannan K. 2017. Mass loading and emission of benzophenone-3 (BP-3) and its derivatives in wastewater treatment plants in New York State, USA. *Sci Total Environ.* 579:1316–1322. doi:10.1016/j.scitotenv.2016.11.124.

Wick A, Fink G, Ternes TA. 2010. Comparison of electrospray ionization and atmospheric pressure chemical ionization for multi-residue analysis of biocides, UV-filters and benzothiazoles in aqueous matrices and activated sludge by liquid chromatography-tandem mass spectrometry. *J Chromatogr A.* 1217(14):2088–2103. doi:10.1016/j.chroma.2010.01.079.

Wu JW, Chen HC, Ding WH. 2013. Ultrasound-assisted dispersive liquid-liquid microextraction plus simultaneous silylation for rapid determination of salicylate and benzophenone-type ultraviolet filters in aqueous samples. *J Chromatogr A.* 1302:20–27. doi:10.1016/j.chroma.2013.06.017.

Wu M hong, Xie D guo, Xu G, Sun R, Xia X yu, Liu W long, Tang L. 2017. Benzophenone-type UV filters in surface waters: An assessment of profiles and ecological risks in Shanghai, China. *Ecotoxicol Environ Saf.* 141:235–241. doi:10.1016/j.ecoenv.2017.03.013.

You L, Nguyen VT, Pal A, Chen H, He Y, Reinhard M, Gin KYH. 2015. Investigation of pharmaceuticals, personal care products and endocrine disrupting chemicals in a tropical urban catchment and the influence of environmental factors. *Sci Total Environ.* 536:955–963. doi:10.1016/j.scitotenv.2015.06.041.

Zhang Q, Ma X, Dzakpasu M, Wang XC. 2017. Evaluation of ecotoxicological effects of benzophenone UV filters: Luminescent bacteria toxicity, genotoxicity and hormonal activity. *Ecotoxicol Environ Saf.* 142:338–347. doi:10.1016/j.ecoenv.2017.04.027.

Zhong, X., Downs, C.A., Che, X., Zhang, Z., Li, Yiman, Liu, B., Li, Q., Li, Yuting, Gao, H. 2019. The toxicological effects of oxybenzone, an active ingredient in suncream personal care products, on prokaryotic alga *Arthrospira* sp. and eukaryotic alga *Chlorella* sp. *Aquat Toxicol*. 216:105295. https://doi.org/10.1016/j.aquatox.2019.105295.

Zeeman M, Gilford J. 1993. Ecological hazard evaluation and risk assessment under EPA’s Toxic Substances Control Act (TSCA): An introduction. In: Environmental Toxicology and Risk Assessment, ASTM STP 1179. p.7-21. Landis WG, Hughes JS, Lewis MA, Eds. Philadelphia, PA: American Society for Testing and Materials. doi: 10.1520/STP19230S.
